# A species-level trait dataset of bats in Europe and beyond

**DOI:** 10.1038/s41597-023-02157-4

**Published:** 2023-05-03

**Authors:** Jérémy S. P. Froidevaux, Nia Toshkova, Luc Barbaro, Ana Benítez-López, Christian Kerbiriou, Isabelle Le Viol, Michela Pacifici, Luca Santini, Clare Stawski, Danilo Russo, Jasja Dekker, Antton Alberdi, Francisco Amorim, Leonardo Ancillotto, Kévin Barré, Yves Bas, Lisette Cantú-Salazar, Dina K. N. Dechmann, Tiphaine Devaux, Katrine Eldegard, Sasan Fereidouni, Joanna Furmankiewicz, Daniela Hamidovic, Davina L. Hill, Carlos Ibáñez, Jean-François Julien, Javier Juste, Peter Kaňuch, Carmi Korine, Alexis Laforge, Gaëlle Legras, Camille Leroux, Grzegorz Lesiński, Léa Mariton, Julie Marmet, Vanessa A. Mata, Clare M. Mifsud, Victoria Nistreanu, Roberto Novella-Fernandez, Hugo Rebelo, Niamh Roche, Charlotte Roemer, Ireneusz Ruczyński, Rune Sørås, Marcel Uhrin, Adriana Vella, Christian C. Voigt, Orly Razgour

**Affiliations:** 1 University of Stirling, Biological and Environmental Sciences, Faculty of Natural Sciences, FK9 4LJ Stirling, UK; 2 Centre d’Ecologie et des Sciences de la Conservation (CESCO, UMR 7204), CNRS, MNHN, Sorbonne-Université, 29900 Concarneau, 75005 Paris, France; 3 School of Biological Sciences, University of Bristol, Life Sciences Building, BS8 1TQ Bristol, UK; 4 Institute of Biodiversity and Ecosystem Research, Bulgarian Academy of Sciences, 1 Tsar Osvoboditel Blvd., 1000 Sofia, Bulgaria; 5 National Museum of Natural History at the Bulgarian Academy of Sciences, Sofia, Bulgaria; 6 DYNAFOR, INRAE-INPT, University of Toulouse, Castanet-Tolosan, France; 7 Integrative Ecology Group, Estación Biológica de Doñana (EBD-CSIC), Sevilla, Spain; 8 Department of Zoology, University of Granada, Granada, Spain; 9 Department of Biology and Biotechnologies “Charles Darwin”, Sapienza University of Rome, Rome, Italy; 10 Department of Biology, Norwegian University of Science and Technology, Trondheim, NO-7491 Norway; 11 School of Science, Technology and Engineering, University of the Sunshine Coast, Maroochydore DC, Queensland 4558 Australia; 12 Laboratory of Animal Ecology and Evolution (AnEcoEvo), Dipartimento di Agraria, Università degli Studi di Napoli Federico II, via Università, 100, 80055 Portici (Napoli), Italy; 13 Jasja Dekker Dierecologie BV, Arnhem, the Netherlands; 14 Center for Evolutionary Hologenomics, Globe Institute, University of Copenhagen, Copenhagen, Denmark; 15 CIBIO, Centro de Investigação em Biodiversidade e Recursos Genéticos, InBIO Laboratório Associado, Campus de Vairão, Universidade do Porto, 4485-661 Vairão, Portugal; 16 BIOPOLIS Program in Genomics, Biodiversity and Land Planning, CIBIO, Campus de Vairão, 4485-661 Vairão, Portugal; 17 CEFE, Univ. Montpellier, CNRS, EPHE, IRD, Montpellier, France; 18 Luxembourg Institute of Science and Technology, Environmental Research and Innovation, 41 rue du Brill, L-4422 Belvaux, Luxemburg; 19 Max Planck Institute of Animal Behavior, Department of Migration, Am Obstberg 1, 78315 Radolfzell, Germany; 20 University of Konstanz, Department of Biology, Universitätsstr. 10, 78464 Konstanz, Germany; 21 Faculty of Environmental Sciences and Natural Resource Management, Norwegian University of Life Sciences, P.O. Box 5003, NO-1432 Ås, Norway; 22 Research Institute of Wildlife Ecology, University of Veterinary Medicine Vienna, Vienna, Austria; 23 Department of Behavioural Ecology, Faculty of Biological Sciences, University of Wroclaw, Sienkiewicza 21, 50-335 Wroclaw, Poland; 24 Ministry of Economy and Sustainable Development, Institute for Environment and Nature, Radnička cesta 80, HR-10000 Zagreb, Croatia; 25 Croatian Biospeleological Society, Rooseveltov trg 6, HR-10000 Zagreb, Croatia; 26 School of Biodiversity, One Health and Veterinary Medicine, College of Medical, Veterinary and Life Sciences, University of Glasgow, Glasgow, G12 8QQ UK; 27 Department Evolutionary Ecology, Estación Biológica de Doñana (EBD-CSIC), Sevilla, Spain; 28 CIBER de Epidemiología y Salud Pública, CIBERESP, 28220 Madrid, Spain; 29 Institute of Forest Ecology, Slovak Academy of Sciences, Zvolen, Slovakia; 30 Mitrani Department of Desert Ecology, Jacob Blaustein Institutes for Desert Research, Ben-Gurion University of the Negev, 8499000 Midreshet Ben-Gurion, Israel; 31 Auddicé Biodiversité– ZAC du Chevalement, 5 rue des Molettes, 59286 Roost-Warendin, France; 32 Institute of Animal Science, Warsaw University of Life Sciences (SGGW), Ciszewskiego 8, 02-787 Warsaw, Poland; 33 Institut de Minéralogie, de Physique des Matériaux et de Cosmochimie (IMPMC), Sorbonne Université, CNRS, MNHN, IRD, 61 Rue Buffon, 75005 Paris, France; 34 Conservation Biology Research Group, Biology Department, University of Malta, MSD2080 Msida, Malta; 35 Institute of Zoology, Chișinău, Republic of Moldova; 36 Technical University of Munich, Terrestrial Ecology Research Group, Department for Life Science Systems, School of Life Sciences, Freising, Germany; 37 ESS, Polytechnic Institute of Setúbal, Campus do IPS - Estefanilha, 2910-761 Setúbal, Portugal; 38 Bat Conservation Ireland, Carmichael House, 4-7, North Brunswick Street, Dublin, D07 RHA8 Ireland; 39 Mammal Research Institute Polish Academy of Sciences, Stoczek 1, 17-230 Białowieża, Poland; 40 Institute of Biology and Ecology, Faculty of Science, P. J, Šafárik University in Košice, Košice, Slovakia; 41 Department Evolutionary Ecology, Leibniz Institute for Zoo and Wildlife Research, Alfred-Kowalke-Str. 17, 10315 Berlin, Germany; 42 Biosciences, University of Exeter, Streatham Campus, Hatherly Laboratories, Prince of Wales Road, Exeter, EX4 4PS UK

**Keywords:** Ecology, Zoology

## Abstract

Knowledge of species’ functional traits is essential for understanding biodiversity patterns, predicting the impacts of global environmental changes, and assessing the efficiency of conservation measures. Bats are major components of mammalian diversity and occupy a variety of ecological niches and geographic distributions. However, an extensive compilation of their functional traits and ecological attributes is still missing. Here we present EuroBaTrait 1.0, the most comprehensive and up-to-date trait dataset covering 47 European bat species. The dataset includes data on 118 traits including genetic composition, physiology, morphology, acoustic signature, climatic associations, foraging habitat, roost type, diet, spatial behaviour, life history, pathogens, phenology, and distribution. We compiled the bat trait data obtained from three main sources: (i) a systematic literature and dataset search, (ii) unpublished data from European bat experts, and (iii) observations from large-scale monitoring programs. EuroBaTrait is designed to provide an important data source for comparative and trait-based analyses at the species or community level. The dataset also exposes knowledge gaps in species, geographic and trait coverage, highlighting priorities for future data collection.

## Background & Summary

Functional traits are becoming increasingly important in large-scale ecological and evolutionary analyses, notably because they allow integrating individual-level information to species level^1^. Functional traits can be defined as any feature measurable at the individual level that can influence fitness^2^. Trait-based approaches functionally link individual organisms to community structure and dynamics through their physiological, morphological, or life-history attributes^3^ and facilitate generalisations across species and their assemblages^4^. Trait-based approaches have become increasingly popular in biogeography^5^, community ecology^1^, macroecology^6^, evolution^7^, conservation biology^8^ and ecosystem functioning^9^. They are now widely used to estimate biodiversity patterns and trends^10,11^, as well as to unveil the mechanisms underlying species assemblages^12^. By understanding how traits covary and are related to environmental variables we can infer general ecological principles that overcome taxonomic gaps. The use of a species-level trait dataset is particularly relevant when working at the community level and in the context of rapid environmental changes.

**Fig. 1:**
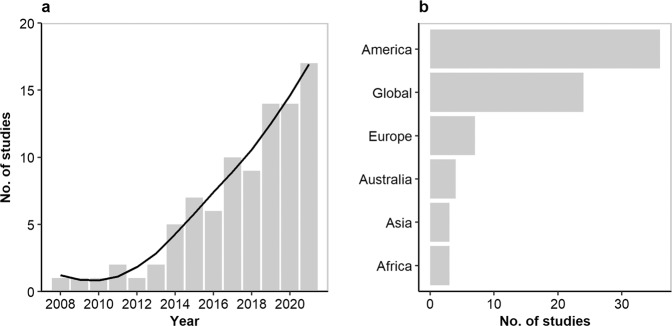
Number of peer-reviewed studies per year (**a**) and per geographic area (**b**) that implemented a trait-based approach to study bats^15–91^. Data were extracted from a systematic literature search conducted in Web of Science and Google Scholar on the 15^th^ of November 2021 using the following search string terms: (Bat* OR Chiroptera) AND (“trait-base*” OR “trait diversity” OR “functional diversity” OR trait*). The black solid line represents the LOESS (locally weighted scatterplot smoothing) fit to the observed relationship. See raw data and details in Supplementary Material 1.

With 1,439 species distributed across the globe^13^, bats (order Chiroptera) account for ca. a fifth of global mammalian diversity. While bats are the second richest taxonomic order of mammals, they have been so far understudied compared to other groups^14^. In the absence of in-depth knowledge of how species respond to environmental gradients, we can make inferences on understudied species using traits of the most closely related taxa. Trait-based approaches are becoming more common in bat research^15–91^ (Fig. 1). For example, Conenna, *et al*.^92^ investigated the traits that favour bat persistence in arid environments across the globe. Similarly, Blakey, *et al*.^93^ investigated traits that explain bat community changes after wildfires. Jung and Threlfall^55^ studied traits relevant for tolerance of urban environments in bats. These studies help overcome the taxonomic knowledge gaps and derive general principles that can explain patterns of bat distribution and abundance, and their responses to the environment. This is especially important in the context of global change, where there is a pressing need for large-scale predictions of biodiversity responses to environmental change^94^.

The application of trait-based approaches in bat research and the inclusion of bats in wider trait-based studies in Europe and elsewhere (Fig. 1) have been limited to date due to lack of relevant and reliable trait data for most bat species. Many European bats have been studied extensively, but the most reliable information on their functional traits has been scattered across the scientific and grey literature and unpublished data held by researchers and special interest groups. There are several ongoing national^95,96^ and regional^97^ bat monitoring programs across Europe that are now old enough to provide highly reliable data on population trends and distributions. These programs also collect systematic and standardised data on morphology, echolocation, hibernation patterns and roost selection that are extremely useful to bat functional ecology.

Here, we present EuroBaTrait 1.0, a comprehensive open-access dataset of 118 traits for 47 European bat species built by compiling data from (i) a systematic literature and dataset search, (ii) unpublished data from pan-European bat experts and (iii) large-scale bat monitoring programs in France. EuroBaTrait 1.0 aims to fill the current knowledge gap to facilitate integrative trait-based evolutionary and ecological research on bat species, and mammals more generally, at the European scale and beyond. As our objective was to build a living dataset, EuroBaTrait 1.0 is intended to be updated annually and its geographic and taxonomic scope can be expanded over time.

## Methods

### Taxon and geographic coverage

Our dataset includes the complete bat fauna of Europe based on the Handbook of the Mammals of Europe^98^. This represents 47 bat species from 12 genera (Table 1). The taxonomic nomenclature follows the Handbook of the Mammals of Europe. The geographic coverage is mainland Europe and European islands, including the British Isles, Mediterranean islands and Macaronesia (Supplementary Material 2). The dataset is based mainly on recent publications, following recent taxonomic revisions (i.e. 2021)^13^

**Table 1:** List of the 47 bat species included in EuroBaTrait 1.0.

Family	Species: scientific name	Species: vernacular name	Taxon URI
Miniopteridae	*Miniopterus schreibersii*	Schreibers’ bent-winged bat	https://www.gbif.org/species/9796816
Molossidae	*Tadarida teniotis*	European free-tailed bat	https://www.gbif.org/species/2433009
Pteropodidae	*Rousettus aegyptiacus*	Egyptian fruit bat	https://www.gbif.org/species/2432953
Rhinolophidae	*Rhinolophus blasii*	Blasius’ horseshoe bat	https://www.gbif.org/species/2432666
Rhinolophidae	*Rhinolophus euryale*	Mediterranean horseshoe bat	https://www.gbif.org/species/2432621
Rhinolophidae	*Rhinolophus ferrumequinum*	Greater horseshoe bat	https://www.gbif.org/species/2432655
Rhinolophidae	*Rhinolophus hipposideros*	Lesser horseshoe bat	https://www.gbif.org/species/2432614
Rhinolophidae	*Rhinolophus mehelyi*	Mehely’s horseshoe bat	https://www.gbif.org/species/2432667
Vespertilionidae	*Barbastella barbastellus*	Barbastelle bat	https://www.gbif.org/species/2432582
Vespertilionidae	*Eptesicus anatolicus*	Anatolian serotine bat	https://www.gbif.org/species/5787592
Vespertilionidae	*Eptesicus bottae*	Botta’s serotine bat	https://www.gbif.org/species/2432346
Vespertilionidae	*Eptesicus isabellinus*	Isabelline serotine bat	https://www.gbif.org/species/5787585
Vespertilionidae	*Eptesicus nilssonii*	Northern bat	https://www.gbif.org/species/7261816
Vespertilionidae	*Eptesicus serotinus*	Common serotine bat	https://www.gbif.org/species/2432359
Vespertilionidae	*Hypsugo savii*	Savi’s pipistrelle	https://www.gbif.org/species/7261861
Vespertilionidae	*Myotis alcathoe*	Alcathoe bat	https://www.gbif.org/species/4266346
Vespertilionidae	*Myotis bechsteinii*	Bechstein’s bat	https://www.gbif.org/species/2432427
Vespertilionidae	*Myotis blythii*	Lesser mouse-eared bat	https://www.gbif.org/species/2432414
Vespertilionidae	*Myotis brandtii*	Brandt’s bat	https://www.gbif.org/species/7261875
Vespertilionidae	*Myotis capaccinii*	Long-fingered bat	https://www.gbif.org/species/2432430
Vespertilionidae	*Myotis crypticus*	Cryptic myotis	https://www.gbif.org/species/9918569
Vespertilionidae	*Myotis dasycneme*	Pond bat	https://www.gbif.org/species/2432452
Vespertilionidae	*Myotis daubentonii*	Daubenton’s bat	https://www.gbif.org/species/2432439
Vespertilionidae	*Myotis davidii*	David’s myotis	https://www.gbif.org/species/4266349
Vespertilionidae	*Myotis emarginatus*	Geoffroy’s bat	https://www.gbif.org/species/2432470
Vespertilionidae	*Myotis escalerai*	Escalera’s bat	https://www.gbif.org/species/8181305
Vespertilionidae	*Myotis myotis*	Greater mouse-eared bat	https://www.gbif.org/species/2432416
Vespertilionidae	*Myotis mystacinus*	Common whiskered bat	https://www.gbif.org/species/9754263
Vespertilionidae	*Myotis nattereri*	Natterer’s bat	https://www.gbif.org/species/2432389
Vespertilionidae	*Myotis punicus*	Maghreb mouse-eared bat	https://www.gbif.org/species/4266337
Vespertilionidae	*Nyctalus azoreum*	Azorean bat	https://www.gbif.org/species/5218523
Vespertilionidae	*Nyctalus lasiopterus*	Greater noctule	https://www.gbif.org/species/5218525
Vespertilionidae	*Nyctalus leisleri*	Leisler’s noctule	https://www.gbif.org/species/5218522
Vespertilionidae	*Nyctalus noctula*	Common noctule	https://www.gbif.org/species/5218524
Vespertilionidae	*Pipistrellus kuhlii*	Kuhl’s pipistrelle	https://www.gbif.org/species/5218464
Vespertilionidae	*Pipistrellus maderensis*	Madeira pipistrelle	https://www.gbif.org/species/5218476
Vespertilionidae	*Pipistrellus nathusii*	Nathusius’ pipistrelle	https://www.gbif.org/species/5218471
Vespertilionidae	*Pipistrellus pipistrellus*	Common pipistrelle	https://www.gbif.org/species/5218465
Vespertilionidae	*Pipistrellus pygmaeus*	Soprano pipistrelle	https://www.gbif.org/species/5707150
Vespertilionidae	*Plecotus auritus*	Brown long-eared bat	https://www.gbif.org/species/5218507
Vespertilionidae	*Plecotus austriacus*	Gray long-eared bat	https://www.gbif.org/species/5739437
Vespertilionidae	*Plecotus gaisleri*	Gaisler’s long-eared bat	https://www.gbif.org/species/10893276
Vespertilionidae	*Plecotus kolombatovici*	Balkan long-eared bat	https://www.gbif.org/species/5739445
Vespertilionidae	*Plecotus macrobullaris*	Alpine long-eared bat	https://www.gbif.org/species/5787719
Vespertilionidae	*Plecotus sardus*	Sardinian long-eared bat	https://www.gbif.org/species/5739436
Vespertilionidae	*Plecotus teneriffae*	Tenerife long-eared bat	https://www.gbif.org/species/5218519
Vespertilionidae	*Vespertilio murinus*	Parti-coloured bat	https://www.gbif.org/species/2432564

### Trait categories

Based on the literature and expert-knowledge, we selected a comprehensive set of relevant traits, including both commonly used and bat-specific traits. These traits reflect a variety of ecological strategies, niches, and functional roles that are routinely collected for bats and other taxa to enable joint analyses with other datasets. Traits were divided into 13 categories: genetic composition (N = 9), physiology (N = 12), morphology (N = 8), acoustic signature (N = 14), climatic associations (N = 4), foraging habitat (N = 29), roost type (N = 12), diet (N = 7), spatial behaviour (N = 7), life history (N = 6), pathogens (N = 3), phenology (N = 2), and distribution (N = 5). While in theory a given trait can fall into different categories, we affiliated each trait to a single, most relevant category for clarity and to simplify analysis.

### Dataset entry

All traits are given at the species and country/European level in the final dataset, but we also provide individual-level data (when available). Each trait value (i.e. dataset entry) is accompanied (whenever possible and applicable) by (i) a location (x,y coordinates and/or country), (ii) contextual characteristics, (iii) source, and (iv) estimates of precision. For aggregated values such as means and medians, we also provide the number of replicates and a measure of dispersion.

### Data sources and acquisition

We followed three strategies to compile the EuroBaTrait 1.0 dataset. First, we conducted a systematic literature and dataset search and retrieved trait data from published books, taxonomic monographs, scientific articles, online resources, and existing datasets. Second, we put out a call to request unpublished data from European bat experts via the network of COST Action Network CA18107 “ClimBats” (https://climbats.eu/). Third, we used data from two large-scale French standardised bat monitoring programs (“Vigie-Chiro” and “CACCHI” programs; https://www.vigienature.fr/fr/chauves-souris & https://croemer3.wixsite.com/teamchiro/cacchi) to retrieve key traits and to derive traits that could not be collected following the first two strategies. The first version of the trait dataset was finalised after the three strategies had been completed for all taxa.

#### Literature and dataset search

We conducted the literature and dataset search using Web of Science and Google Scholar. We implemented a multi-step approach to target our search. In step 1, we based our search at the trait category level using the search string “(Bat* OR Chiroptera)” in addition to the name of a given trait category (see section 2.2 Trait categories) (e.g. “(Bat* OR Chiroptera)” and “acoustic*”). In step 2, we looked for each specific trait name (e.g. “(Bat* OR Chiroptera)” and “call duration”). In step 3, we focused our search on missing data. For this we used as a search string the name of the missing trait and the Latin name of the target bat species (e.g. “*Plecotus auritus*” and “call duration”). Altogether, we included trait data from six published books, nine taxonomic monographs, 378 scientific articles (published in indexed journals that were checked using clarivate https://mjl.clarivate.com/), 84 articles from the grey literature, 19 unpublished data sources, and three datasets (AnAge^99^, date of access: 02/02/2021, IUCN^100^, date of access: 20/01/2021; DBatVir^101^, date of access: 18/01/2021).

#### ClimBats COST Action network

Morphological measures taken from bats captured for research and monitoring are rarely published, so we asked European bat experts to provide data for traits that are usually not found in the literature. We contacted experts through the ClimBats network (representing 28 countries and ca. 100 experts), organised meetings to explain the project goals, and asked for raw or summarised unpublished trait data (e.g. morphological measurements). Data were also provided by the UK Bat Conservation Trust National Bat Monitoring Programme (https://www.bats.org.uk/our-work/national-bat-monitoring-programme). In total, we retrieved 279 trait values (i.e. dataset entry) for two morphological traits (mean forearm length and body mass) measured on 37 species in 12 countries (Bulgaria, Germany, Ireland, Italy, Luxembourg, Moldova, Norway, Poland, Portugal, Slovakia, Spain and the United Kingdom).

#### Large-scale bat monitoring programs

We used “CACCHI” (capture data) and the French national-scale citizen-science bat monitoring program “Vigie-Chiro” (acoustic data) which are coordinated by the French National Museum of Natural History (MNHN) to retrieve data on key traits and identify missing ones. CACCHI is a French bat monitoring program that aims to achieve national consistency regarding technical and ethical aspects of capture practices and data collection. French bat workers contribute to this program by providing data collected in a local context, thus giving the opportunity to build a dataset on a large spatio-temporal scale. We retrieved four traits from CACCHI, including mean forearm length and body mass (>60,000 and >6,000 dataset entries, respectively). Vigie-Chiro is one of the largest citizen-science bat monitoring programs in Europe with a standardized stationary point survey (among three) corresponding to a total of 16,349 sites acoustically monitored during 2015–2021 by >500 participants (see an overall description of Vigie-Chiro in Supplementary Material 3). We retrieved 33 species-level traits from the data collected by Vigie-Chiro, including acoustic signature, climatic associations, foraging habitat, and phenology (see full details in Supplementary Material 3). In brief, traits related to acoustic signature (i.e. buzz duration, buzz peak frequency, buzz rate, call duration, call maximum/minimum frequency, call frequency at half call duration, call peak frequency, call slope and interpulse interval) were directly extracted from the reference library of calls using TADARIDA software^102^. For traits related to climatic associations (i.e. responses to nightly temperature, precipitation, and wind speed) and foraging habitats (i.e. responses to deciduous forest, coniferous forest, dense urban area, freshwater, cropland and grassland at three spatial scales (50 m, 500 m and 5000 m radius buffer scale)), we used data from the stationary point protocol. We conducted a series of univariate generalized linear mixed models (‘glmmTMB’ R package^103^) with species-specific bat activity as response variables and foraging habitat and weather conditions as explanatory ones. The latter were standardized (mean = 0, SD = 1) and we extracted the slope of the relationship to inform the trait value. Finally, we derived the traits related to phenology (Kurtosis index and Skewness index of the seasonal activity pattern) with the ‘moments’ R package^104^, which required plotting bat activity obtained from the stationary point protocol as a function of Julian day.

## Data Records

The EuroBaTrait 1.0 dataset is available at figshare^105^ and through the R Shiny App (https://jasja.shinyapps.io/ClimBats/) under the terms of a Creative Commons Attribution 4.0 International waiver. The CC-BY-4.0 waiver facilitates the discovery, re-use, and citation of the dataset. As this is a dynamic and living dataset, future updates of the dataset will be made directly on figshare and associated R Shiny App. We will follow the same technical validation as described hereafter.

### Dataset structure

We followed the general recommendations proposed by Schneider, *et al*.^106^ to organise the dataset. The dataset consists of 16 tables linked by unique identifiers. The “Taxon” table includes the full scientific name and family of all taxa and a uniform resource identifier linked to GBIF (https://www.gbif.org/, Table 1). The “Trait description” table represents the metadata, in which we describe the different traits (name, category, unit, data type) and define each trait following (when available) current glossaries (e.g. we followed Blatteis, *et al*.^107^ for physiology). The “Trait reference” table lists the full references used to build the dataset. The dataset also includes one table per trait category – namely, genetic composition, physiology, morphology, acoustic signature, climatic associations, foraging habitat, roost type, diet, spatial behaviour, life history, pathogens, phenology, and distribution – with the core observation values (SD, CV and N referring to standard deviation, coefficient of variation and sample size, respectively) and associated information. We decided not to impute missing values to highlight research gaps and data needs.

### Dataset completeness

**Fig. 2:**
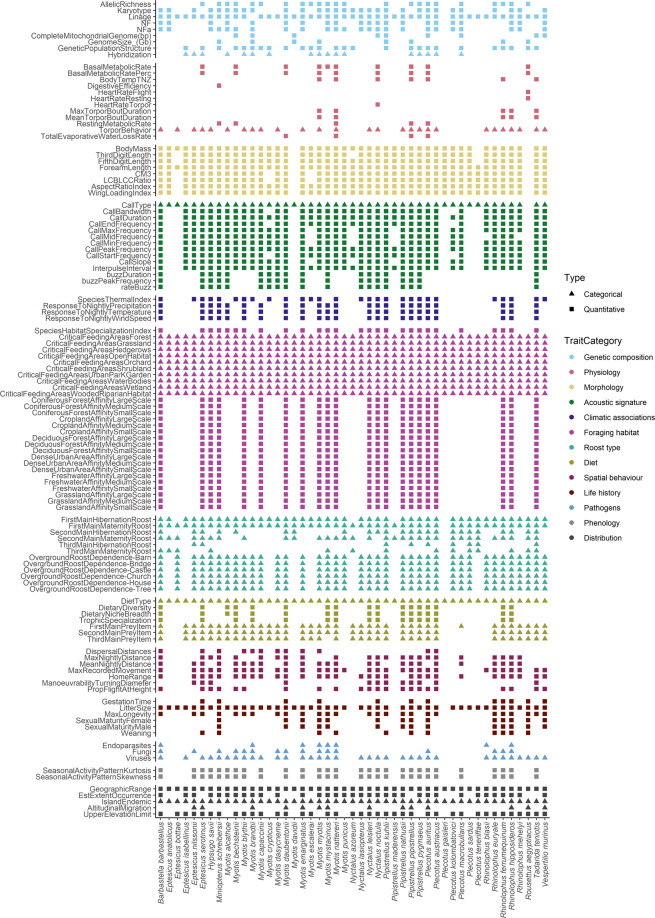
Trait by species matrix illustrating the EuroBaTrait 1.0 dataset completeness.

**Fig. 3:**
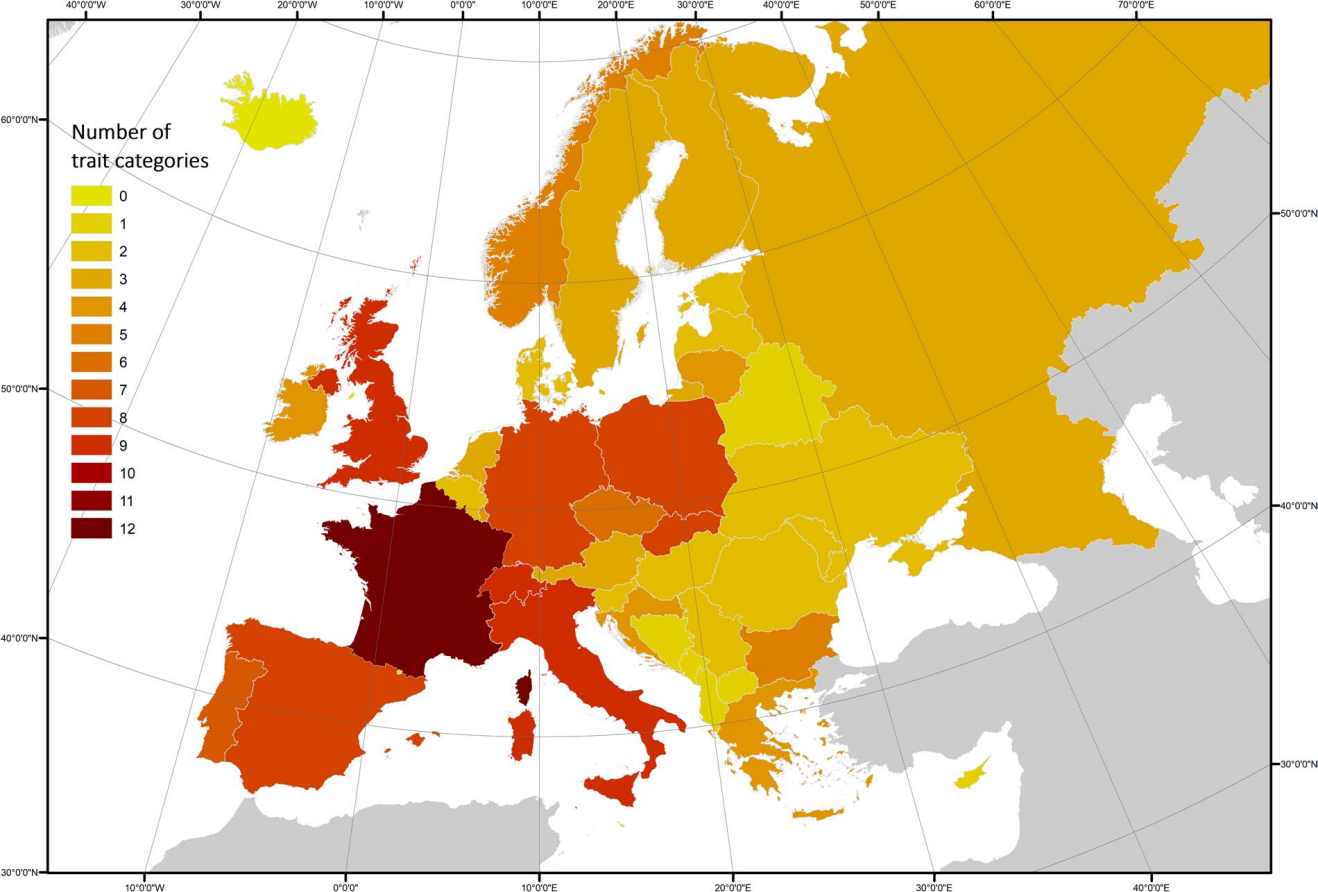
Number of trait categories (genetic composition, physiology, morphology, acoustic signature, climatic associations, foraging habitat, roost type, diet, spatial behaviour, life history, pathogens, phenology, and distribution) provided at the country level across all species. For sake of clarity and for highlighting gaps in geographic coverage, we did not consider in this map traits provided across a given species’ range or at regional level. Details on each trait category are provided in Supplementary Material 4.

Assessing dataset completeness is crucial for identifying knowledge gaps and highlighting future data collection priorities. The overall completeness of the dataset can be assessed based on species coverage, geographic coverage, and trait resolution. The EuroBaTrait 1.0 dataset has broad taxonomic coverage (Fig. 2). At the trait category level, the species coverage of trait information is (i) complete or nearly complete (i.e. at least one trait from these categories encompasses >95% of the species) for foraging habitat (100%), diet (100%), distribution (100%), acoustic signature (98%), roost type (98%), genetic composition (96%) and morphology (96%), (ii) moderately complete (between 75% and 95%) for life history (94%) and pathogens (72%), and (iii) at a low level of completeness (<75%) for spatial behaviour (66%), climatic associations (60%), phenology (60%) and physiology (55%). Note that the number of traits documented differ greatly amongst trait categories (see section 2.2 Trait categories and Fig. 2). At the trait level, species coverage ranges from 100% (e.g. geographic range in distribution) to 2% (e.g. heart rate during flight or rest in physiology). The physiology category has the lowest number of traits covered per species (Fig. 2) and it is evident that more research effort is needed to improve our knowledge on bat physiology. Regardless of trait type, we lack information on the four island endemic species (*Nyctalus azoreum*, *Pipistrellus maderensis*, *Plecotus sardus*, and *Plecotus teneriffae*) with >75% of traits missing for these species. Regarding geographic coverage, there is a clear longitudinal bias for many trait categories with most data originating from Western Europe (Fig. 3). Nevertheless, this bias is more evident for some traits (e.g. foraging habitat and spatial behaviour) than others (e.g. roost type and pathogens, Supplementary Material 4) because few traits and species have been comprehensively measured in many locations throughout Europe. Finally, when looking at the trait resolution, quantitative data (continuous and discrete) were available for 71% of the traits. This proportion, however, largely varies between trait categories (e.g. 0% for roost and 100% for morphology). Increasing species and geographic coverage as well as trait resolution (from categorical to quantitative data, whenever possible) remains a real challenge that needs to be overcome to implement a more robust trait-based approach^108^. To that end, enhancing collaboration among researchers/practitioners is the way forward, as witnessed here with the collection of the largest morphological dataset on bats in Europe if not in the world (measurements on body mass of ca. 55,000 individuals from 37 species and forearm length of ca. 120,000 individuals from 39 species).

#### Current limitations

We acknowledge that many traits could not be compiled for the EuroBaTrait dataset 1.0 at their finest resolution. This is particularly the case for the diet trait category. There are many published and unpublished studies in different languages depicting European bat diets using traditional (e.g. microscopic faecal or stomach content analysis) or molecular (e.g. DNA metabarcoding) methods. However, gathering such a large amount of information at the highest resolution was not feasible at this stage, and we aim to provide a much more comprehensive treatment of this trait category in future releases of the EuroBaTrait dataset.

Trait quality, here defined as the degree of confidence in a trait value, is another important component to consider when assessing dataset completeness. However, evaluation of trait quality is subject to a certain degree of subjectivity. To allow future users of the dataset to evaluate trait information quality, we provide for each trait value the original sources and contextual characteristics that may include (when available) sample size (e.g. number of individuals) associated with the trait values. For quantitative continuous data, we also derived the coefficient of variation as a standardised measure of dispersion of the values around the mean.

### Data visualization

We created a R Shiny app to help the users visualizing the trait data. It is freely accessible at the following URL: https://jasja.shinyapps.io/ClimBats/.

## Technical Validation

We employed two main strategies to ensure the accuracy in the data included in the EuroBaTrait 1.0 dataset. First, we looked for erroneous data in the dataset using a series of plots (e.g. boxplots and frequency histograms to detect potential outliers across species in continuous data) alongside traditional statistical validation techniques (e.g. outlier test) in R v4.1.1^109^. When an outlier was detected, we looked at the original sources and cross-checked with other sources (e.g. Handbook of the Mammals of Europe) the plausibility and reliability of the observation. Second, we asked several experts to conduct data quality control and error detection in their field of expertise. Experts on a given bat species revised the trait associated with that species, while experts on a given trait category revised all traits from that category. This allowed us to cross validate the full dataset. Furthermore, as we provide the original references for each trait value collected during the literature and dataset search and describe in detail the methods used for the traits computed with data from the large-scale bat monitoring programs, users can assess the validity and accuracy of the original sources (note that >75% of our data sources are peer-reviewed articles published in indexed scientific journals) and method used. Finally, we strongly encourage users to report any errors and additional data sources directly to the corresponding authors. As it is a living dataset, the inclusion of new data will rigorously follow the same technical validation as described above.

## Usage Notes

### Overview

**Fig. 4:**
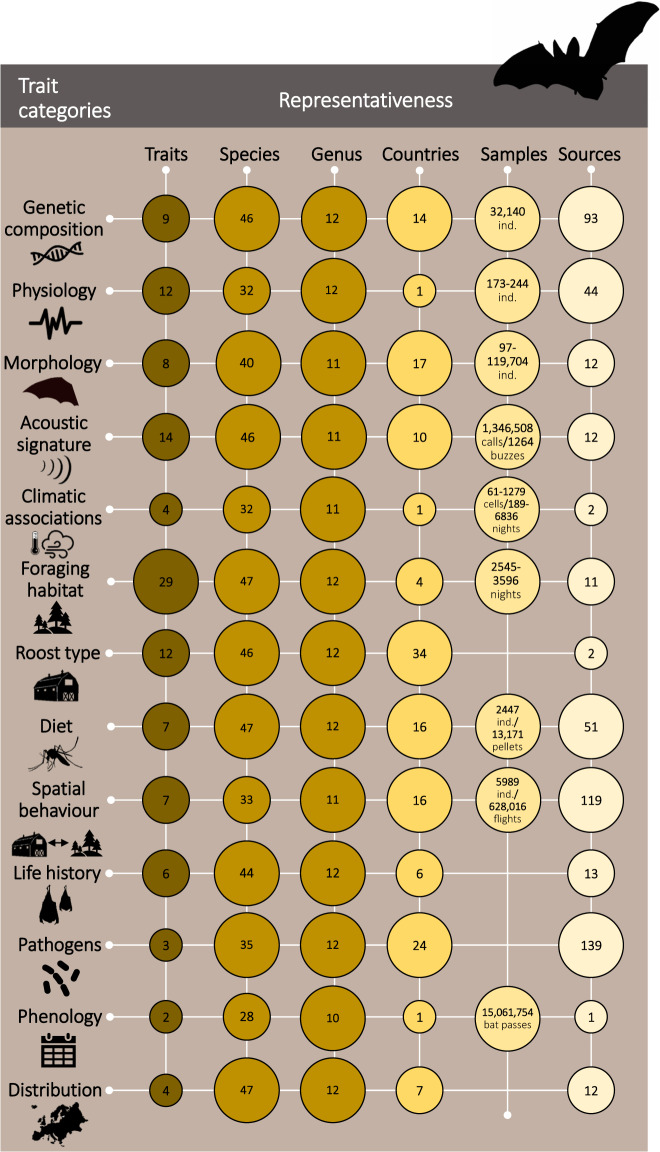
Representativeness of the EuroBaTrait 1.0 dataset. Summary of the 13 trait categories included in the trait dataset and their coverage in terms of number of traits included under the category, number of species and genera with data for at least one of the traits under the category, number of countries with data for at least one trait under the category (regional or global data were not included in these calculations), number of samples (individuals, calls or nights) used to generate values for the traits, and number of data sources on which the trait values are based.

Here we present the first comprehensive dataset of functional traits in European bats (Fig. 4). The dataset has numerous applications in community ecology, macroecology, biogeography, conservation biology, ecophysiology, evolutionary biology, and virology/epidemiology for species and/or community approaches. For example, the dataset enables researchers to investigate functional redundancy and complementarity of coexisting bat species, to establish linkages among functional traits, as well as between functional traits and ecosystem functioning, and to assess trait-environment relationships and their impacts on bat distributional patterns^92^. This in turn is relevant for addressing relevant questions in conservation and global change biology. The paucity of trait data on bats has, until now, prevented the testing of large-scale correlations between the intrinsic characteristics of species and major drivers of decline in this taxon^11,110,111^. The EuroBaTrait dataset will contribute to assessing and quantifying the role of each trait in determining bats’ risk of extinction.

EuroBaTrait is also designed to be used to investigate functional responses of bat assemblages to various environmental drivers of change and their impacts on ecosystem functions and services^5^. This could be done by relating environmental variables or processes to traits, i.e. either by calculating complementary and integrative functional diversity metrics such as functional richness, evenness or dispersion^112^ or by computing individual Community-Weighted Mean trait values^113^. Such trait-based approaches allow the inference of several key properties of bat communities including effective functional originality based on both rarity and distinctiveness of species in functional trait spaces to better support bat conservation programmes^114,115^. Three-table ordination methods can also be used to directly link bat trait syndromes to spatial or temporal environmental gradients (e.g.^43–48^). These trait-based analyses constitute a methodological corpus that is still seldom used by bat ecologists, while it can provide a relevant standard analysis approach to study the response of bat functional diversity and trait assemblages to bioclimatic variables, or environmental degradation and conservation measures (Fig. 1; see for example^17,20,30,32,38,41,51,67,83,89,116^). Finally, bats are increasingly acknowledged for performing key ecological functions in semi-natural habitats as well as in production forests and agroecosystems, that ultimately provide valuable ecosystem services^117–120^. As a result, the functional trait-based characterisation of bat communities to determine their role in ecological networks will greatly benefit from the EuroBaTrait dataset in future bat ecological research.

### From a static to a dynamic trait dataset

The data descriptor was peer reviewed in 2023 based on the data available on the platform at the time^105^. It is our intention to keep improving and enriching the dataset over time, so while the paper presents version 1, new versions may be released in the future. For this reason, we invite anyone to (i) share their data in a standardized way via the R Shiny app at https://jasja.shinyapps.io/ClimBats/ and (ii) cite the dataset stored at figshare according to the specific version used as well as this publication, when using all or part of the dataset. We also encourage the wider bat research community to build on this first version and engage in updating it via meetings and workshops during dedicated bat congress (e.g. European Bat Research Symposiums).

### Towards an open access individual-level trait dataset

We encourage researchers and practitioners to collect, store publicly and publish trait values at the individual level. As most functional traits tend to vary greatly among individuals according to age, sex, condition, state, gene pool and behavioural personality^121^, this individual-level variation may persist across time and space and upscale at the local community level (e.g. mean individual specialisation or mean home range size and composition)^122,123^. Natural populations of a given species are composed of sets of individuals that occupy subsets of the species’ niche. For instance, several studies of bats provide evidence of individual specialisation in diet^124^. Furthermore, trait-based approaches accounting for within-individual variation in species traits can inform us about the role of phenotypic plasticity in species’ responses to anthropogenic threats and drivers of global change (e.g.^125–127^). Within-individual data are particularly valuable in seasonal species like bats, which have to cope with reproduction, hibernation and/or migration. While we acknowledge that some traits can only be measured at the species level, the lack of individual-level trait information we observed during data collection likely arises from the fact that: (i) only summarised information (e.g. mean/median, accompanied with an estimate of precision at best) has traditionally been provided in the published literature, (ii) researchers/practitioners and/or institutions have limited time and resources, as well as almost no formal recognition (although the situation is now improving in many countries), for publishing such data^128^. We provide in Supplementary Material 5 trait information collected at the individual level, but we strongly encourage the bat expert community and associated institutions to share and open their data (FAIR Data principles) to enable the progression from a species-level trait dataset to an individual-level trait dataset.

## Supplementary information

A species-level trait dataset of bats in Europe and beyond -- Supplementary Material

A species-level trait dataset of bats in Europe and beyond -- Supplementary Material

## Data Availability

The code to create the R Shiny App is available on GitHub (https://github.com/J4SJA/ClimBatsApp).
