# Supplementary material for: A species-level trait dataset of bats in Europe and beyond
Source: Sci Data. 2023 May 3;10:253. doi: 10.1038/s41597-023-02157-4 (PMC10156679; doi:10.1038/s41597-023-02157-4)
Supplement: Supplementary file 1 — A species-level trait dataset of bats in Europe and beyond -- Supplementary Material [file 41597_2023_2157_MOESM1_ESM.docx]

**A species-level trait dataset of bats in Europe and beyond**

**--**

**Supplementary Material**

[Supplementary Material 1. Literature review 2](#_Toc131148985)

[Supplementary Material 2. Geographic coverage of EuroBaTrait 1.0 8](#_Toc131148986)

[Supplementary Material 3. Description of the French national-scale citizen-science bat monitoring program Vigie-Chiro and associated traits 9](#_Toc131148987)

[Supplementary Material 4. Geographic coverage of each trait category 13](#_Toc131148988)

[Supplementary Material 5. Individual-level morphological traits for France 17](#_Toc131148989)

Supplementary Material 1. Literature review

**Table 1.** List of peer-reviewed studies that implemented a trait-based approach to study bats. Data were extracted from a systematic literature search conducted in Web of Science and Google Scholar on the 15^th^ of November 2021 using the following search string terms: (Bat* OR Chiroptera) AND ("trait-base*" OR "trait diversity" OR "functional diversity" OR trait*).

| **ID** | **1st author** | **title** | **journal** | **DOI** | **year of publication** | **geographic area** | **bat versus multi–taxa** | **No. of bat species** |
| --- | --- | --- | --- | --- | --- | --- | --- | --- |
| 1 | Alberdi | DNA metabarcoding and spatial modelling link diet diversification with distribution homogeneity in European bats | Nature Communications | 10.1038/s41467-020-14961-2 | 2020 | Europe | bat | 7 |
| 2 | Bader | Mobility explains the response of aerial insectivorous bats to anthropogenic habitat change in the Neotropics | Biological Conservation | 10.1016/j.biocon.2015.02.028 | 2015 | Panama | bat | 13 |
| 3 | Barbaro | Biotic predictors complement models of bat and bird responses to climate and tree diversity in European forests | Proceedings of the Royal Society B | 10.1098/rspb.2018.2193 | 2019 | Europe | multi-taxa | 27 |
| 4 | Becker | Ecological and evolutionary drivers of haemoplasma infection and bacterial genotype sharing in a Neotropical bat community | Molecular Ecology | 10.1111/mec.15422 | 2019 | Belize | bat | 33 |
| 5 | Belmaker | Relative roles of ecological and energetic constraints, diversification rates and region history on global species richness gradients | Ecology Letters | 10.1111/ele.12438 | 2015 | world | multi-taxa | / |
| 6 | Blakey | Terrestrial laser scanning reveals below-canopy bat trait relationships with forest structure | Remote Sensing of Environment | 10.1016/j.rse.2017.05.038 | 2017 | Australia | bat | 13 |
| 7 | Bogoni | Interacting elevational and latitudinal gradients determine bat diversity and distribution across the Neotropics | Journal of Animal Ecology | 10.1111/1365-2656.13594 | 2021 | America | bat | >300 |
| 8 | Bowler | A cross-taxon analysis of the impact of climate change on abundance trends in central Europe | Biological Conservation | 10.1016/j.biocon.2015.03.034 | 2015 | Europe | multi-taxa | 11 |
| 9 | Burns | Correlates of dispersal extent predict the degree of population genetic structuring in bats | Conservation Genetics | 10.1007/s10592-014-0623-y | 2014 | world | bat | 43 |
| 10 | Byamungu | Abiotic and biotic drivers of functional diversity and functional composition of bird and bat assemblages along a tropical elevation gradient | Diversity and Distributions | 10.1111/ddi.13403 | 2021 | Tanzania | multi-taxa | 20 |
| 11 | Carrasco-Rueda | Dimensions of phyllostomid bat diversity and assemblage composition in a tropical forest-agricultural landscape | Diversity | 10.3390/d12060238 | 2020 | Peru | bat | 43 |
| 12 | Carstens | A global analysis of bats using automated comparative phylogeography uncovers a surprising impact of Pleistocene glaciation | Journal of Biogeography | 10.1111/jbi.13382 | 2018 | world | bat | 302 |
| 13 | Carvalho | Patterns and drivers determining phyllostomid bat diversity in land-bridge islands off the south-east coast of Brazil | Biological Journal of the Linnean Society | 10.1093/biolinnean/blab112 | 2021 | Brazil | bat | / |
| 14 | Carvalho | Drafting a blueprint for functional and phylogenetic diversity conservation in the Brazilian Cerrado | Natureza & Conservação | 10.4322/natcon.00802011 | 2010 | Brazil | multi-taxa | / |
| 15 | Carvalho | Taxonomic, functional and phylogenetic bat diversity decrease from more to less complex natural habitats in the Amazon | Oecologia | 10.1007/s00442-021-05009-3 | 2021 | Brazil | bat | 47 |
| 16 | Carvalho | Traits that allow bats of tropical lowland origin to conquer mountains: Bat assemblages along elevational gradients in the South American Atlantic Forest | Journal of Biogeography | 10.1111/jbi.13506 | 2019 | Brazil | bat | 35 |
| 17 | Charbonnier | Bat and bird diversity along independent gradients of latitude and tree composition in European forests | Oecologia | 10.1007/s00442-016-3671-9 | 2016 | Europe | multi-taxa | 26 |
| 18 | Cisneros | Effects of human-modified landscapes on taxonomic, functional and phylogenetic dimensions of bat biodiversity | Diversity and Distributions | 10.1111/ddi.12277 | 2015 | Costa-Rica | bat | 34 |
| 19 | Cisneros | Environmental and spatial drivers of taxonomic, functional, and phylogenetic characteristics of bat communities in human-modified landscapes | PeerJ | 10.7717/peerj.2551 | 2016 | Costa-Rica | bat | 34 |
| 20 | Cisneros | Multiple dimensions of bat biodiversity along an extensive tropical elevational gradient | Journal of Animal Ecology | 10.1111/1365-2656.12201 | 2014 | Peru | bat | 92 |
| 21 | Collen | Investing in evolutionary history: implementing a phylogenetic approach for mammal conservation | Philosophical Transactions of the Royal Society B | 10.1098/rstb.2011.0109 | 2011 | world | multi-taxa | / |
| 22 | Cooke | Projected losses of global mammal and bird ecological strategies | Nature Communications | 10.1038/s41467-019-10284-z | 2019 | world | multi-taxa | / |
| 23 | Cox | Diel niche variation in mammals associated with expanded trait space | Nature Communications | 10.1038/s41467-021-22023-4 | 2021 | world | multi-taxa | / |
| 24 | Duchamp | Shifts in bat community structure related to evolved traits and features of human-altered landscapes | Landscape Ecology | 10.1007/s10980-008-9241-8 | 2008 | USA | bat | 8 |
| 25 | Farneda | Trait-related responses to habitat fragmentation in Amazonian bats | Journal of Applied Ecology | 10.1111/1365-2664.12490 | 2015 | Brazil | bat | 26 |
| 26 | Farneda | Predicting biodiversity loss in island and countryside ecosystems through the lens of taxonomic and functional biogeography | Ecography | 10.1111/ecog.04507 | 2020 | Brazil and Panama | bat | 48 |
| 27 | Farneda | Functional recovery of Amazonian bat assemblages following secondary forest succession | Biological Conservation | 10.1016/j.biocon.2017.12.036 | 2018 | Brazil | bat | 46 |
| 28 | Farneda | Effects of land-use change on functional and taxonomic diversity of Neotropical bats | Biotropica | 10.1111/btp.12736 | 2019 | America | bat | 103 |
| 29 | Frank | Phylogeny, traits, and biodiversity of a neotropical bat assemblage: close relatives show similar responses to local deforestation | The American Naturalist | 10.1086/692534 | 2017 | Costa-Rica | bat | 42 |
| 30 | Garcia-Herrera | Functional traits of bats associated with the use of wetlands in Colombian tropical dry forests | Acta Chiropterologica | 10.3161/15081109ACC2020.22.2.005 | 2020 | Colombia | bat | / |
| 31 | García-Morales | Deforestation impacts on bat functional diversity in tropical landscapes | PLoS ONE | 10.1371/journal.pone.0166765 | 2016 | Mexico | bat | 12 |
| 32 | González-Maya | Spatial patterns of species richness and functional diversity in Costa Rican terrestrial mammals: implications for conservation | Diversity and Distributions | 10.1111/ddi.12373 | 2016 | Costa-Rica | multi-taxa | / |
| 33 | Grilo | Roadkill risk and population vulnerability in European birds and mammals | Frontiers in Ecology and the Environment | 10.1002/fee.2216 | 2020 | Europe | multi-taxa | / |
| 34 | Guy | Phylogeny matters: revisiting ‘a comparison of bats and rodents as reservoirs of zoonotic viruses | Royal Society Open Science | 10.1098/rsos.181182 | 2019 | world | multi-taxa | 1150 |
| 35 | Guy | The influence of bat ecology on viral diversity and reservoir status | Ecology and Evolution | 10.1002/ece3.6315 | 2020 | world | bat | 812 |
| 36 | Han | Undiscovered bat hosts of filoviruses | PLoS Neglected Tropical Diseases | 10.1371/journal.pntd.0004815 | 2016 | world | bat | 1116 |
| 37 | Hanspach | Using trait-based filtering as a predictive framework for conservation: a case study of bats on farms in southeastern Australia | Journal of Applied Ecology | 10.1111/j.1365-2664.2012.02159.x | 2012 | Australia | bat | 10 |
| 38 | Herrera | Disassembly of fragmented bat communities in Orange Walk District, Belize | Acta Chiropterologica | 10.3161/15081109ACC2018.20.1.011 | 2018 | Belize | bat | 32 |
| 39 | Holt | Environmental variation is a major predictor of global trait turnover in mammals | Journal of Biogeography | 10.1111/jbi.13091 | 2017 | world | multi-taxa | / |
| 40 | Jakobsson | Contrasting multi-taxa functional diversity patterns along vegetation structure gradients of woody pastures | Biodiversity and Conservation | 10.1007/s10531-020-02037-y | 2020 | Sweden | multi-taxa | 10 |
| 41 | Jung | Trait-dependent tolerance of bats to urbanization: a global meta-analysis | Proceedings of the Royal Society B | 10.1098/rspb.2018.1222 | 2018 | world | bat | 180 |
| 42 | Kamilar | Connecting proximate mechanisms and evolutionary patterns: pituitary gland size and mammalian life history | Journal of Evolutionary Biology | 10.1111/jeb.12715 | 2015 | world | multi-taxa | 17 |
| 43 | Kellner | Niche breadth and vertebrate sensitivity to habitat modification: signals from multiple taxa across replicated landscapes | Biodiversity and Conservation | 10.1007/s10531-019-01785-w | 2019 | USA | multi-taxa | 7 |
| 44 | Kosman | Severe limitations of the FEve metric of functional evenness and some alternative metrics | Ecology and Evolution | 10.1002/ece3.6974 | 20220 | Peru | multi-taxa | 5 |
| 45 | Laurindo | Drivers of bat roles in Neotropical seed dispersal networks: abundance is more important than functional traits | Oecologia | 10.1007/s00442-020-04662-4 | 2020 | neotropical region | bat | 46 |
| 46 | Lentini | A global synthesis of survival estimates for microbats | Biology Letters | 10.1098/rsbl.2015.0371 | 2015 | world | bat | 44 |
| 47 | Luis | A comparison of bats and rodents as reservoirs of zoonotic viruses: are bats special? | Proceedings of the Royal Society B | 10.1098/rspb.2012.2753 | 2013 | world | multi-taxa | 1150 |
| 48 | Magg | Faunal surrogates for forest species conservation: A systematic niche-based approach | Ecological Indicators | 10.1016/j.ecolind.2019.01.084 | 2019 | Germany | multi-taxa | / |
| 49 | Mancini | Different bat guilds have distinct functional responses to elevation | Acta Oecologica | 10.1016/j.actao.2019.03.004 | 2019 | Brazil | bat | 22 |
| 50 | Martínez-Ferreira | Taxonomic and functional diversity and composition of bats in a regenerating neotropical dry forest | Diversity | 10.3390/d12090332 | 2020 | Mexico | bat | 15 |
| 51 | Mazel | The geography of ecological niche evolution in mammals | Current Biology | 10.1016/j.cub.2017.03.046 | 2017 | world | multi-taxa | / |
| 52 | Melo | treeNODF: nestedness to phylogenetic, functional and other tree-based diversity metrics | Methods in Ecology and Evolution | 10.1111/2041-210X.12185 | 2014 | Caribbean | bat | / |
| 53 | Moir | Functional diversity and trait filtering of insectivorous bats relate to forest biogeography and fragmentation in South Africa | Journal of Biogeography | 10.1111/jbi.14069 | 2021 | South Africa | bat | 21 |
| 54 | Mollentze | Viral zoonotic risk is homogenous among taxonomic orders of mammalian and avian reservoir hosts | Proceedings of the National Academy of Sciences | 10.1073/pnas.1919176117 | 2020 | world | multi-taxa | / |
| 55 | Monadjem | Species richness patterns and functional traits of the bat fauna of arid southern Africa | Hystrix, the Italian Journal of Mammalogy | 10.4404/hystrix–00016-2017 | 2018 | Southern Africa | bat | 17 |
| 56 | Núñez | Echolocation and stratum preference: key trait correlates of vulnerability of insectivorous bats to tropical forest fragmentation | Frontiers in Ecology and Evolution | 10.3389/fevo.2019.00373 | 2019 | Brazil | bat | 19 |
| 57 | Olival | Host and viral traits predict zoonotic spillover from mammals | Nature | 10.1038/nature22975 | 2017 | world | multi-taxa | / |
| 58 | Peñaranda | Predicting and setting conservation priorities for Bolivian mammals based on biological correlates of the risk of decline | Conservation Biology | doi.org/10.1111/cobi.12453 | 2014 | Bolivia | multi-taxa | 89 |
| 59 | Penone | Global mammal beta diversity shows parallel assemblage structure in similar but isolated environments | Proceedings of the Royal Society B | 10.1098/rspb.2016.1028 | 2016 | world | multi-taxa | / |
| 60 | Phelps | Assemblage and species threshold responses to environmental and disturbance gradients shape bat diversity in disturbed cave landscapes | Diversity | 10.3390/d10030055 | 2018 | Philippines | bat | 21 |
| 61 | Pineda | Frog, bat, and dung beetle diversity in the cloud forest and coffee agroecosystems of Veracruz, Mexico | Conservation Biology | 10.1111/j.1523-1739.2005.00531.x | 2004 | Mexico | multi-taxa | / |
| 62 | Presley | Phylogenetic and functional underdispersion in Neotropical phyllostomid bat communities | Biotropica | 10.1111/btp.12501 | 2018 | Costa-Rica | bat | 33 |
| 63 | Ramírez-Mejía | Functional diversity of phyllostomid bats in an urban–rural landscape: A scale-dependent analysis | Biotropica | 10.1111/btp.12816 | 2020 | Colombia | bat | 20 |
| 64 | Rossoni | A multiple peak adaptive landscape based on feeding strategies and roosting ecology shaped the evolution of cranial covariance structure and morphological differentiation in phyllostomid bats | Evolution | 10.1111/evo.13715 | 2019 | world | bat | 48 |
| 65 | Scheiner | Decomposing functional diversity | Methods in Ecology and Evolution | 10.1111/2041-210X.12696 | 2017 | Peru | bat | 92 |
| 66 | Stevens | Relative contributions of ecological drift and selection on bat community structure in interior Atlantic Forest of Paraguay | Oecologia | 10.1007/s00442-020-04683-z | 2020 | Paraguay | bat | 9 |
| 67 | Stobo-Wilson | Sharing meals: Predation on Australian mammals by the introduced European red fox compounds and complements predation by feral cats | Biological Conservation | 10.1016/j.biocon.2021.109284 | 2021 | Australia | multi-taxa | 80 |
| 68 | Thaxter | Bird and bat species’ global vulnerability to collision mortality at wind farms revealed through a trait-based assessment | Proceedings of the Royal Society B | 10.1098/rspb.2017.0829 | 2017 | world | multi-taxa | 31 / 881 |
| 69 | Threlfall | Ecological processes in urban landscapes: mechanisms influencing the distribution and activity of insectivorous bats | Ecography | 10.1111/j.1600-0587.2010.06939.x | 2011 | Australia | bat | 17 |
| 70 | Tucios-Casco | Ecological gradients explain variation of phyllostomid bat (Chiroptera: Phyllostomidae) diversity in Honduras | Mammalian Biology | 10.1007/s42991-021-00152-z | 2021 | Honduras | bat | 40 |
| 71 | Turmelle | Correlates of viral richness in bats (order Chiroptera) | EcoHealth | 10.1007/s10393-009-0263-8 | 2009 | world | bat | 33 |
| 72 | Walsh | A preliminary ecological profile of Kyasanur Forest disease virus hosts among the mammalian wildlife of the Western Ghats, India | Ticks and Tick-borne Diseases | 10.1016/j.ttbdis.2020.101419 | 2020 | India | multi-taxa | 7 |
| 73 | Wells | Distinct spread of DNA and RNA viruses among mammals amid prominent role of domestic species | Global Ecology and Biogeography | 10.1111/geb.13045 | 2019 | world | multi-taxa | / |
| 74 | Willoughby | A comparative analysis of viral richness and viral sharing in cave-roosting bats | Diversity | 10.3390/d9030035 | 2017 | world | bat | 205 |
| 75 | Wordley | Bats in the Ghats: Agricultural intensification reduces functional diversity and increases trait filtering in a biodiversity hotspot in India | Biological Conservation | 10.1016/j.biocon.2017.03.026 | 2017 | India | bat | 16 |
| 76 | Worsley-Tonks | Using host traits to predict reservoir host species of rabies virus | PLoS Neglected Tropical Diseases | 10.1371/journal.pntd.0008940 | 2020 | world | multi-taxa | 326 |
| 77 | Zamora-Gutierrez | Vulnerability of bat–plant pollination interactions due to environmental change | Global Change Biology | 10.1111/gcb.15611 | 2021 | Mexico | bat | 12 |

Note: Studies using guild or functional groups or community mean trait based (e.g. community specialization index) as response variables were not included (even though these groups were based on traits).

Supplementary Material 2. Geographic coverage of EuroBaTrait 1.0

**
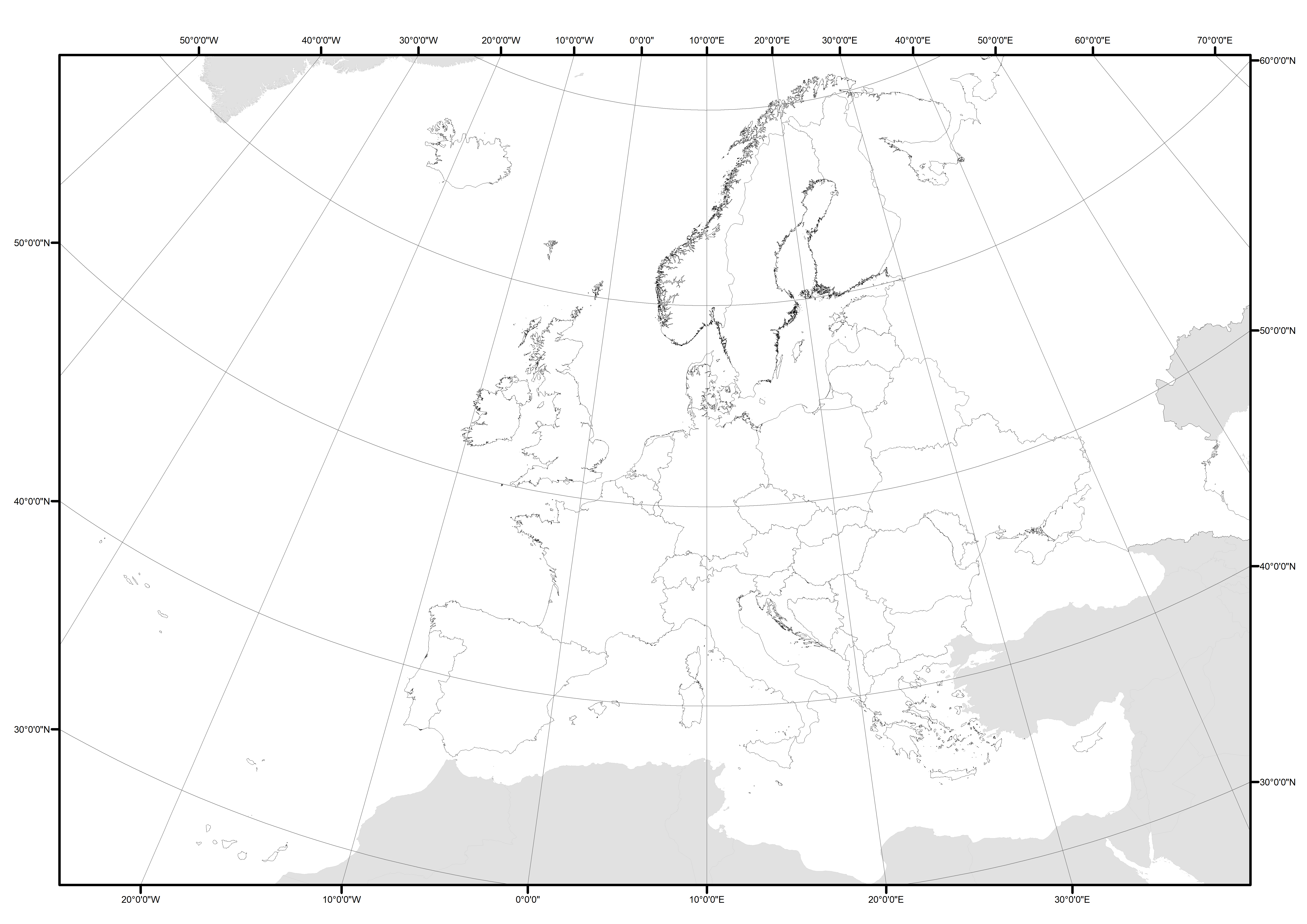
**

**Figure 1**. Map of the geographic coverage of EuroBaTrait 1.0. The geographic coverage is mainland Europe and European islands, including the British Isles, Mediterranean islands and Macaronesia.

Supplementary Material 3. Description of the French national-scale citizen-science bat monitoring program Vigie-Chiro and associated traits

The French national-scale citizen-science bat monitoring program ‘Vigie-Chiro’ has been created and coordinated by the French National Museum of Natural History since 2006. There are three main acoustic monitoring-based protocols proposed to volunteers: the car transect protocol, the walk transect protocol and the stationary point protocol. Hereafter, only data from the stationary point protocol were considered. For this protocol, volunteers set stationary recording devices to record sounds between 8 and 192 kHz throughout the entire night, from 30 min before sunset to 30 min after sunrise (Dubos et al. 2021). The Vigie-Chiro program was originally designed to study bat population trends in France. One major prerequisite to meet such an objective is to ensure that the proportion of habitats sampled is largely representative of French land-use. Thus, volunteers taking part in the stationary point protocol are encouraged to survey randomly selected sampling points, even though this protocol also offers flexibility for volunteers to focus on particular sites. Volunteers are invited to geolocate their device in a systematic national 2-km square grid with several options: (i) volunteers can choose the square monitored or (ii) used a randomly chosen square in a radius of 10km from their home, (i.e. on average one square randomly chosen between 80 possible squares). In addition, within each square volunteers can (i) choose the site monitored or (ii) used a predefined site. Regardless of the option chosen, sampling points should be located at least 200 meters from each other. Preliminary analysis showed that Vigie-Chiro sites covered similar land-use type gradients as sites randomly selected in France (see Mariton et al. 2022). Volunteers may use a variety of full-spectrum ultrasound recorders, including Song Meter SM2BAT+, SM3BAT, SM4BAT, SMminiBAT (Wildlife Acoustics Inc., Concord, MA, USA), Batlogger (Elekon, Luzern, Switzerland), Passive Recorder (https://framagit.org/PiBatRecorderPojects/TeensyRecorders), Anabat Swift (Titley Scientific, Brendale, Australia) and AudioMoth (Hill et al. 2018), as long as the device settings meet the Vigie-Chiro criteria (e.g. standardised trigger setting to limit heterogeneity in detectability). Sampling sessions occurred whenever possible when weather conditions were favourable, i.e. no rain, temperature above seasonal normal and avoiding strong winds (<30 km.h-1), even though long-term monitoring over consecutive nights did not always meet these criteria. Volunteers are encouraged to monitor bats at least twice a year: a first visit during June and July, when females are expected to give birth and feed their offspring; and a second visit between 15 August and 31 September, when juveniles are flying and migratory adults are expected to be contacted. Once the field recording is over, volunteers upload raw acoustic data on the Vigie-Chiro platform. Data uploaded are then automatically processed with the TADARIDA bat identification software (Bas et al. 2017). This software automatically detects and extracts sound parameters of recorded echolocation calls and classifies them into known classes (i.e bat taxa) according to a confidence index value using random forest algorithm (Breiman 2001). For methods to account for identification errors in acoustic surveys, see Barré et al. (2019).

**Acoustic signature**

*Computed traits: buzz duration, buzz peak frequency, buzz rate, call duration, call maximum/minimum frequency, call frequency at half call duration, call peak frequency, call slope and interpulse interval.*

We used the extensive library of reference bat echolocation calls (N sequences = 8737; N calls = 207,742) implemented in the TADARIDA software for identifying bat species in the recordings collected through Vigie-Chiro and compute the 10 acoustic traits.

**Foraging habitats**

*Computed traits: Responses to deciduous forest, coniferous forest, dense urban area, freshwater, cropland and grassland at three spatial scales (50 m, 500 m and 5000 m radius buffer scale).*

We used data from the stationary points protocol (extraction 2020-10-15). We only retained species that were present in at least 300 sites. We considered all bat passes with an identification success probability above 50%. We focused on sites that were in metropolitan France and away from roosts. We focused on nights monitored between May and October and between 2014 to 2020. We calculated the amount of deciduous forest, coniferous forest, dense urban area, freshwater, cropland and grassland cover at three spatial scales around the sampling sites (50 m, 500 m and 5000 m radius buffer scale) using the CES OSO land cover data 2018 (10 m resolution, https://www.theia-land.fr/ceslist/ces-occupation-des-sols/).

We conducted a series of univariate generalized linear mixed models (GLMMs, R package ‘glmmTMB’, Brooks et al. (2017)) to model independently the relationships between species-specific bat activity and each landscape variable measured at different spatial scales. We considered bat activity (i.e. the total number of passes of a given species recorded during a night) as response variable, landscape metrics and Julian day as explanatory variables, and site ID as random effects to avoid pseudoreplication when multiple nights of recordings occurred. Models were fitted with a negative binomial distribution to handle overdispersion. Landscape variables and Julian day were standardized prior inclusion to the models. We hence performed 18 models for each bat species and extracted the estimate (and its standard error) of the modelled relationship.

**Phenology**

*Computed traits: Kurtosis index and Skewness index of the seasonal activity pattern*

We used data from the stationary points protocol (extraction 2021-03-12). For each species we only retained nights for which there was at least one pass of the species with an identification success probability above 90%. We then considered only bat passes with an identification success probability above 50%. We focused on sites that were in metropolitan France, away from roosts and below 500 m below sea level to avoid any bias due to alpine context. We focused on nights monitored between May and August and between 2014 to 2020.

We plotted bat activity (i.e. the total number of passes of a given species recorded during a night) as a function of Julian day and extracted using the ‘moments’ R package (Komsta and Novomestky 2015) the Kurtosis and Skewness values.

**Climatic associations**

*Computed traits: Responses to nightly temperature, precipitation, and wind speed*

We used data from the stationary points protocol (extraction 2020-11-03). For each species we only retained nights for which there was at least one pass of the species with an identification success probability above 90%. We then considered only bat passes with an identification success probability above 50%. We focused on sites that were in metropolitan France, away from roosts and below 500 m below sea level to avoid any bias due to alpine context. We focused on nights monitored between May and August and between 2014 to 2020.

For each night monitored, we extracted precipitations (in mm) and temperature (in °C) from the EObs daily gridded observational dataset which has a high temporal resolution (daily) and a high spatial resolution (0.1 deg) (Cornes et al. 2018) (Copernicus, https://surfobs.climate.copernicus.eu/dataaccess/access_eobs.php#datafiles, R package ‘ncdf4’).

Each monitored site was associated with the grid cell whose centre was closer. For each night, the sum of precipitations and the mean temperature during the day when the night began were extracted. We used the R package ‘RNCEP’ (Kemp et al. 2012) to extract wind speed data from the NCEP/NCAR Reanalysis dataset (Kalnay et al. 1996). Data spatial resolution was 2.5 deg, and each monitored site was associated with the grid cell whose centre was closer. Data temporal resolution was high (6 hours), we extracted data at 6:00 PM the day the monitored night began. We defined wind speed as follows:

Equation 1: Wind_speed= √( (U_wind_component)^2 + (V_wind_component)^2 )

with: U_wind_component : U-wind component (East/West) in m.s-1; and V_wind_component : V-wind component (North/South) in m.s-1.

We conducted a series of univariate generalized linear mixed models (GLMMs, R package ‘glmmTMB’, Brooks et al. (2017)) to model independently the influence of weather conditions on species-specific bat activity. We considered bat activity (i.e. the total number of passes of a given species recorded during a night) as response variable, weather metrics (temperature, precipitation and wind speed) as explanatory variables, and site ID as random effects to avoid pseudoreplication when multiple nights of recordings occurred. Models were fitted with a negative binomial distribution to handle overdispersion. Weather variables were standardized prior inclusion to the models. We hence performed three models for each bat species and extracted the estimate (and its standard error) of the modelled relationship.

Finally, it is important to highlight that because surveys mainly took place during suitable meteorological conditions for bats to forage (as recommended by the Vigie-Chiro guidelines), we did not explore the full gradient of meteorological conditions, with unsuitable conditions (e.g. high wind speed, low temperature) being underrepresented.

**References**

Barré, K., Le Viol, I., Julliard, R., Pauwels, J., Newson, S.E., Julien, J.F., Claireau, F., Kerbiriou, C., Bas, Y., 2019. Accounting for automated identification errors in acoustic surveys. Methods in Ecology and Evolution 10, 1171-1188.

Bas, Y., Bas, D., Julien, J.-F., 2017. Tadarida: A toolbox for animal detection on acoustic recordings. Journal of open research software 5, 6.

Breiman, L., 2001. Random forests. Machine learning 45, 5-32.

Brooks, M.E., Kristensen, K., van Benthem, K.J., Magnusson, A., Berg, C.W., Nielsen, A., Skaug, H.J., Machler, M., Bolker, B.M., 2017. glmmTMB balances speed and flexibility among packages for zero-inflated generalized linear mixed modeling. The R journal 9, 378-400.

Cornes, R.C., van der Schrier, G., van den Besselaar, E.J., Jones, P.D., 2018. An ensemble version of the E‐OBS temperature and precipitation data sets. Journal of Geophysical Research: Atmospheres 123, 9391-9409.

Dubos, N., Kerbiriou, C., Julien, J.-F., Barbaro, L., Barré, K., Claireau, F., Froidevaux, J., Le Viol, I., Lorrillière, R., Roemer, C., Verfaillie, F., Bas, Y., 2021. Going beyond species richness and abundance: robustness of community specialisation measures in short acoustic surveys. Biodiversity and Conservation 30, 343-363.

Hill, A.P., Prince, P., Piña Covarrubias, E., Doncaster, C.P., Snaddon, J.L., Rogers, A., 2018. AudioMoth: Evaluation of a smart open acoustic device for monitoring biodiversity and the environment. Methods in Ecology and Evolution 9, 1199-1211.

Kalnay, E., Kanamitsu, M., Kistler, R., Collins, W., Deaven, D., Gandin, L., Iredell, M., Saha, S., White, G., Woollen, J., 1996. The NCEP/NCAR 40-year reanalysis project. Bulletin of the American meteorological Society 77, 437-472.

Kemp, M.U., Van Loon, E.E., Shamoun-Baranes, J., Bouten, W., 2012. RNCEP: global weather and climate data at your fingertips. Methods in Ecology & Evolution 3, 65-70.

Komsta, L., Novomestky, F., 2015. Moments, cumulants, skewness, kurtosis and related tests. R package version 14.

Mariton, L., Kerbiriou, C., Bas, Y., Zanda, B., Le Viol, I., 2022. Even low light pollution levels affect the spatial distribution and timing of activity of a “light tolerant” bat species. Environmental Pollution 305, 119267.

Supplementary Material 4. Geographic coverage of each trait category


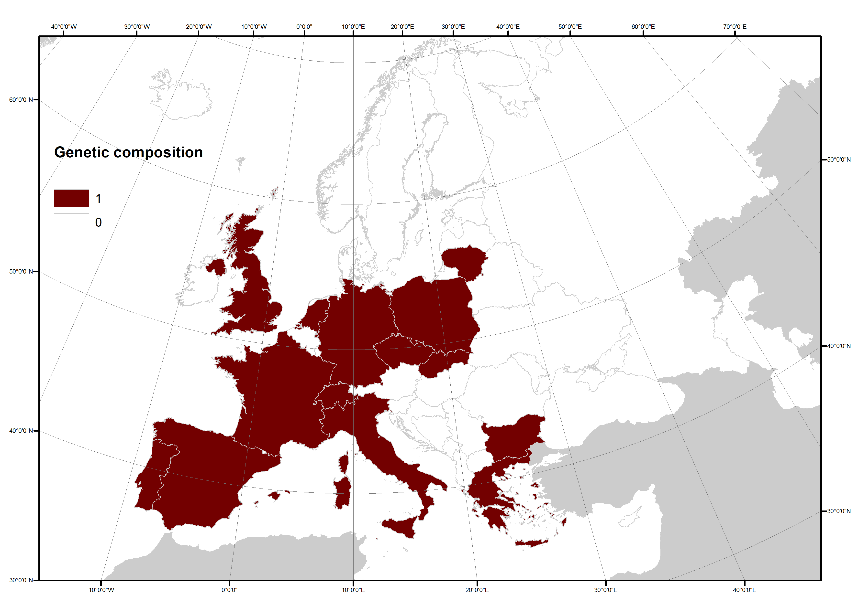

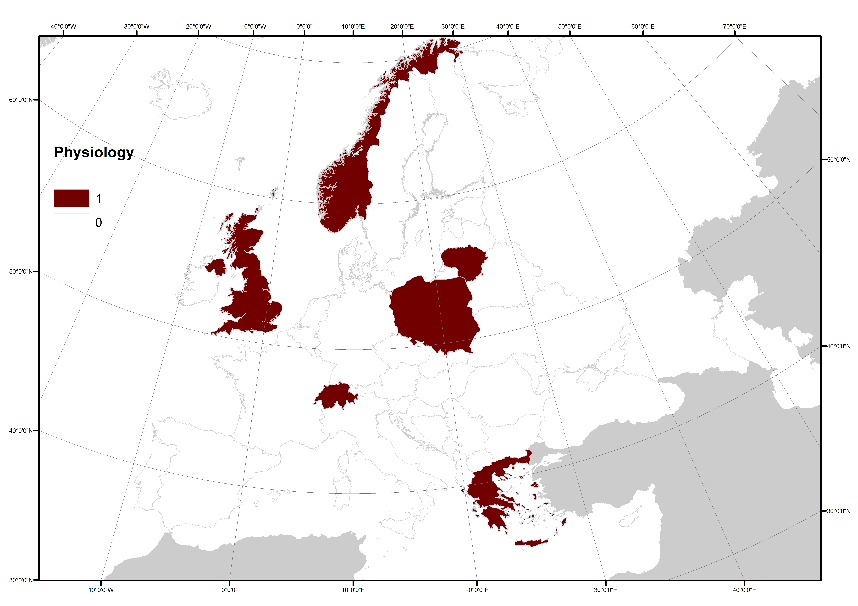

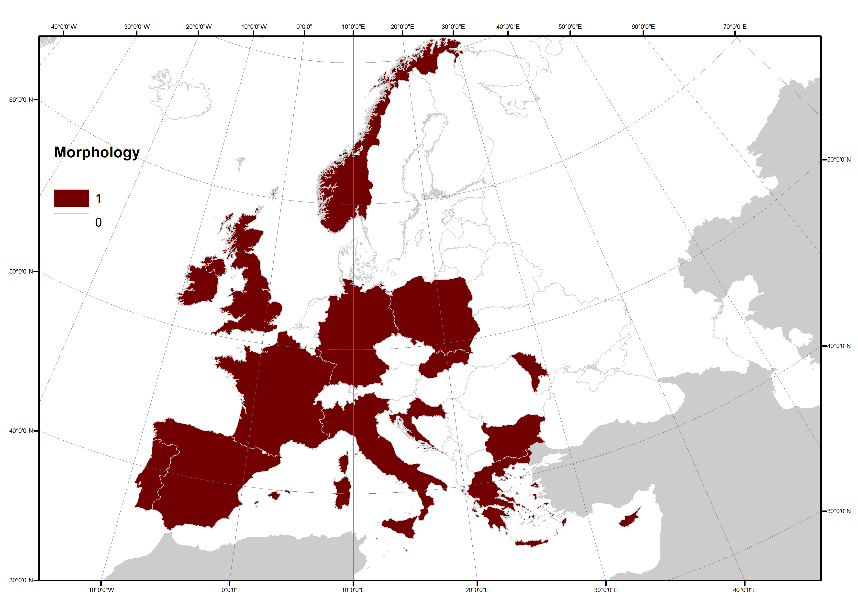

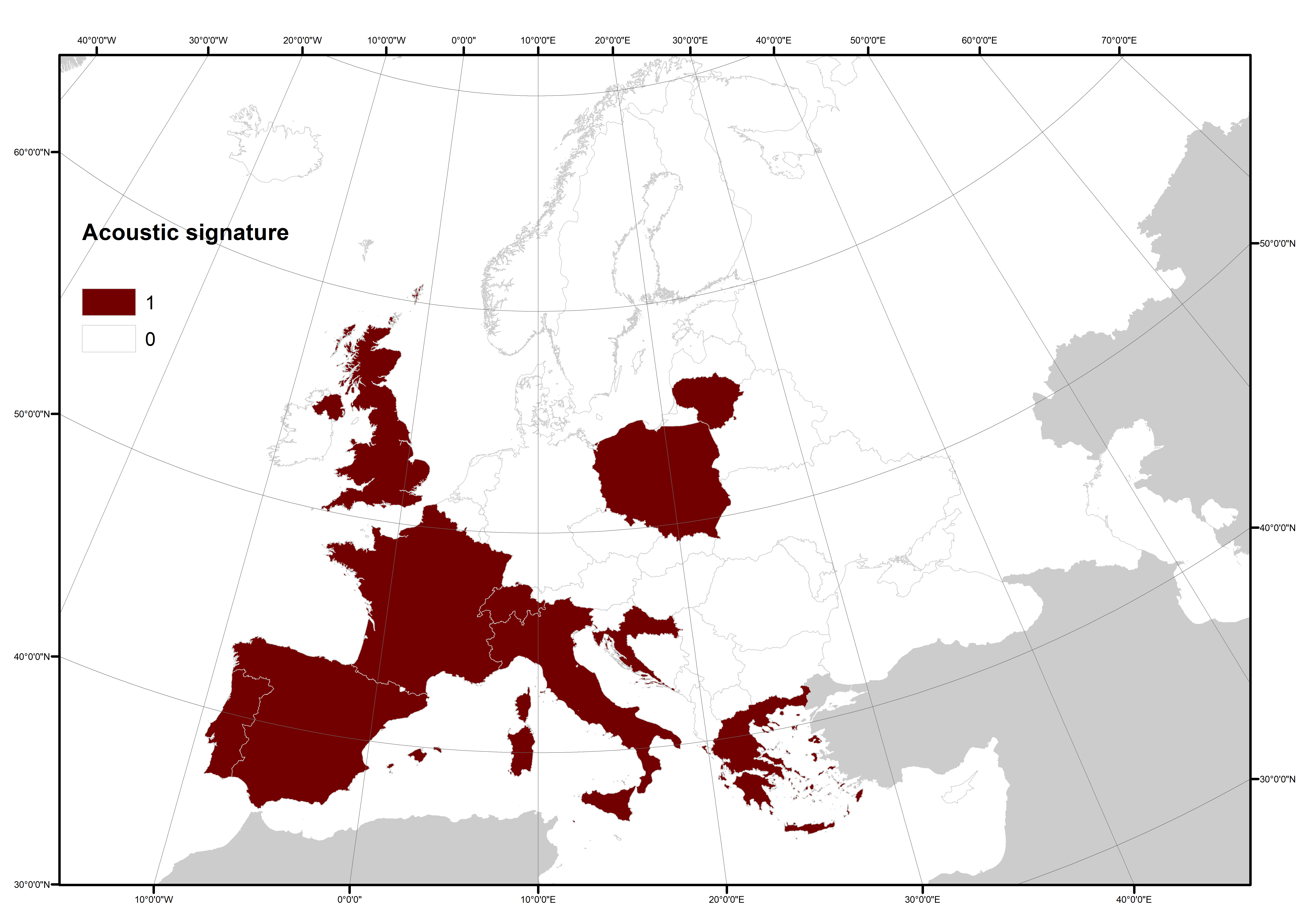


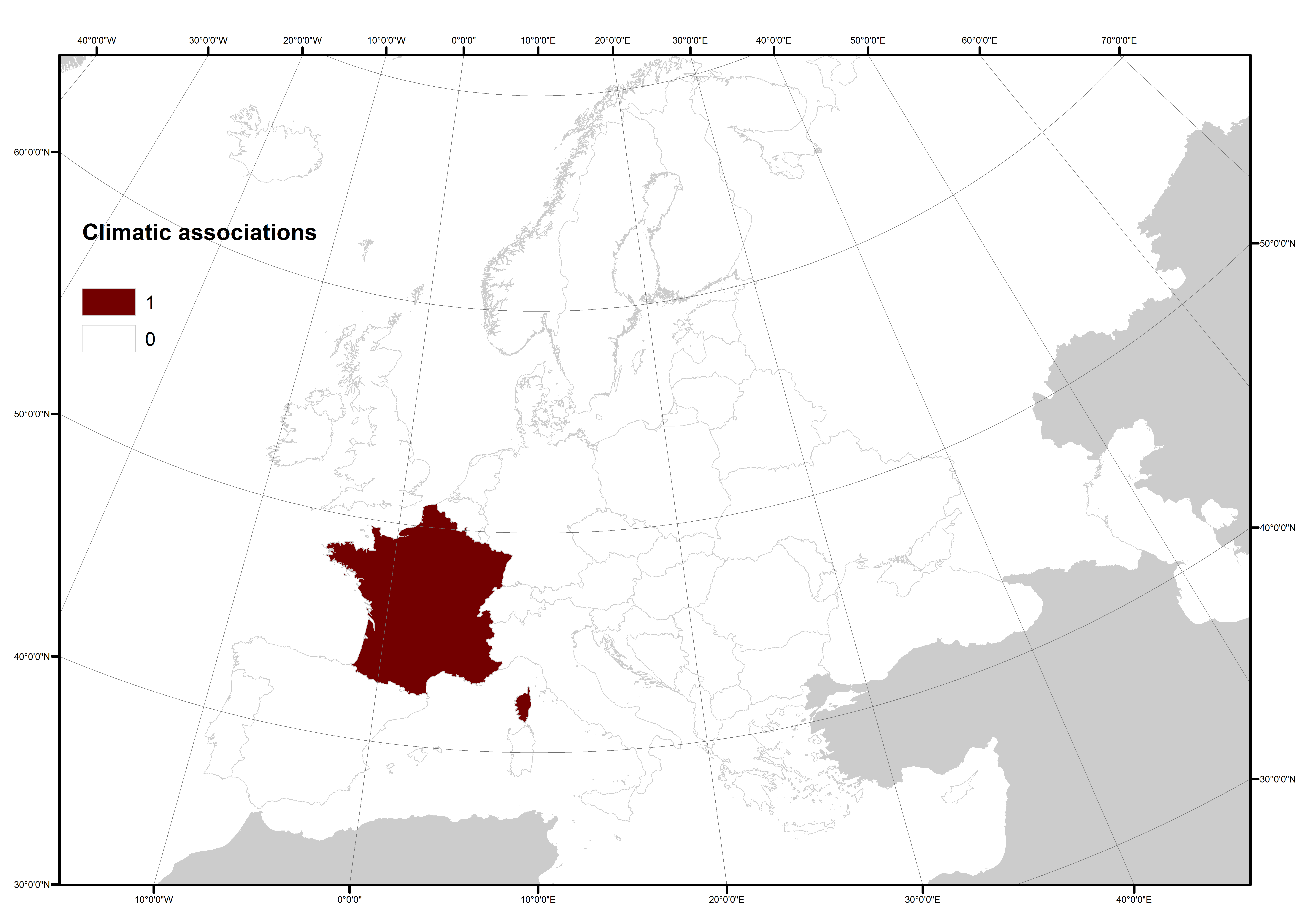

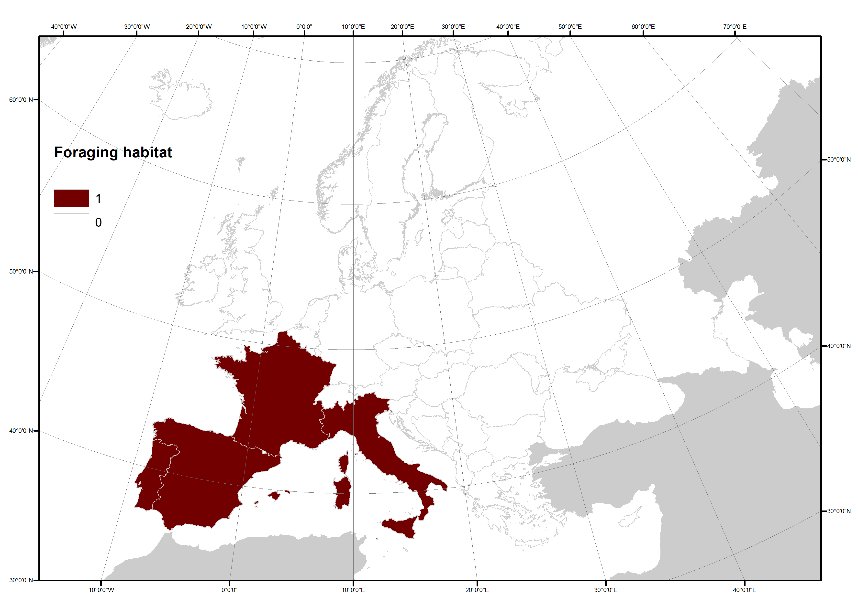

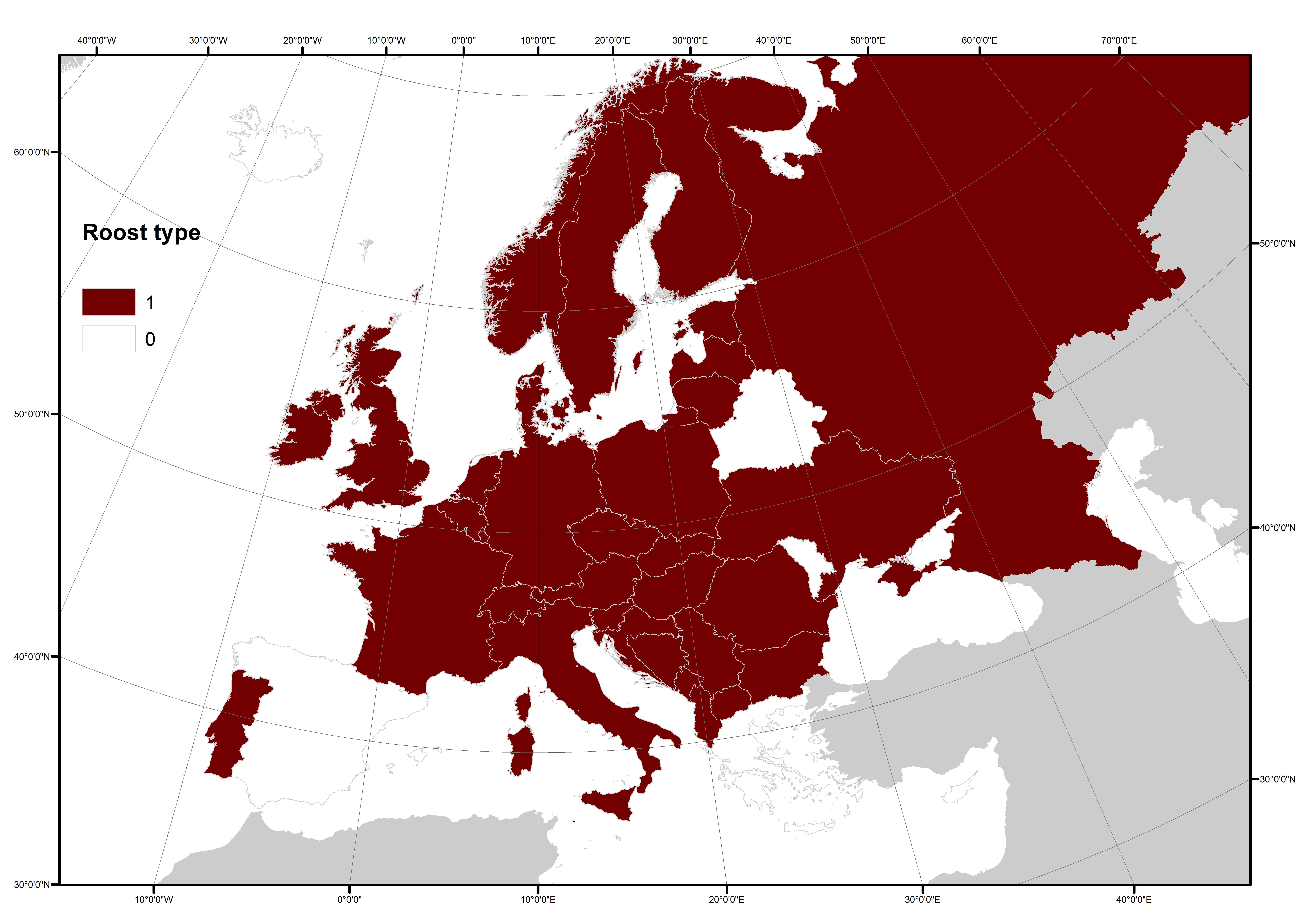

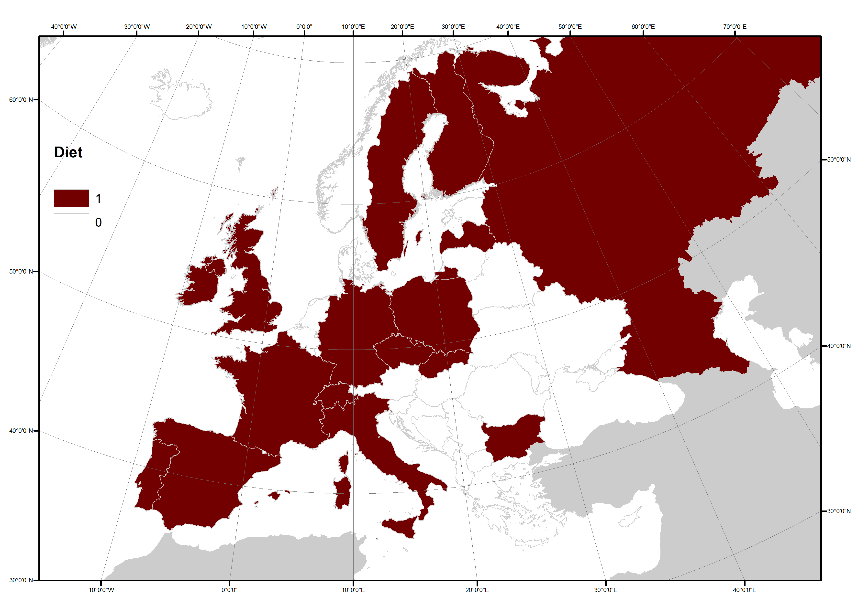

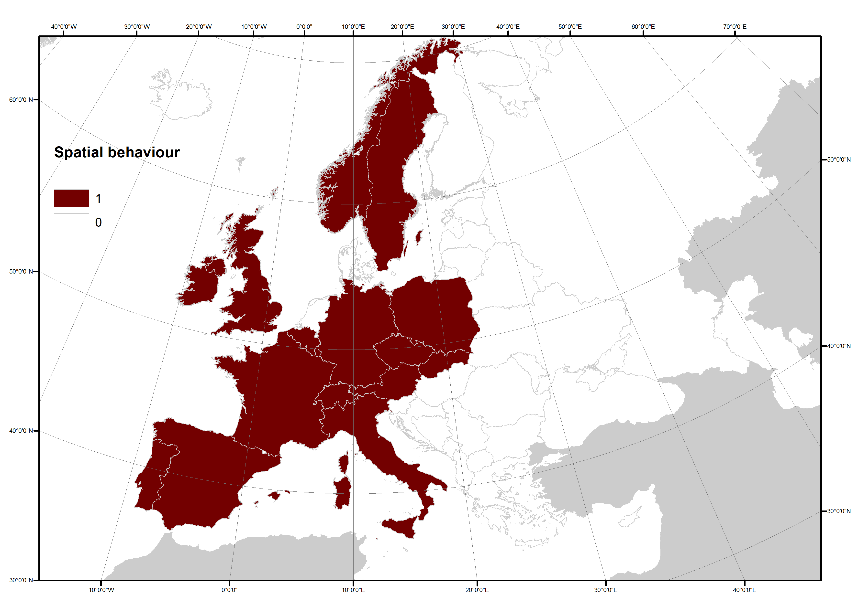

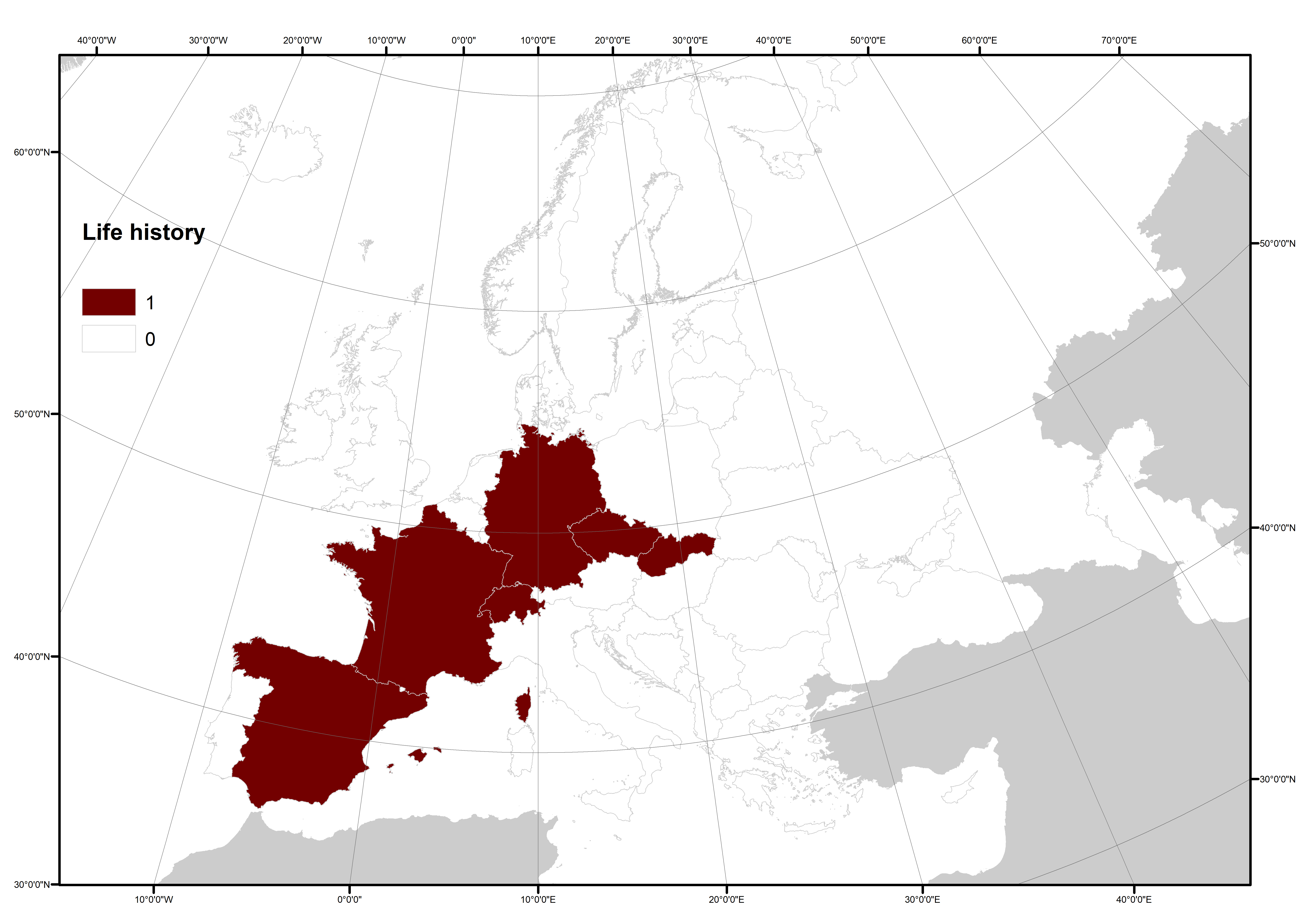

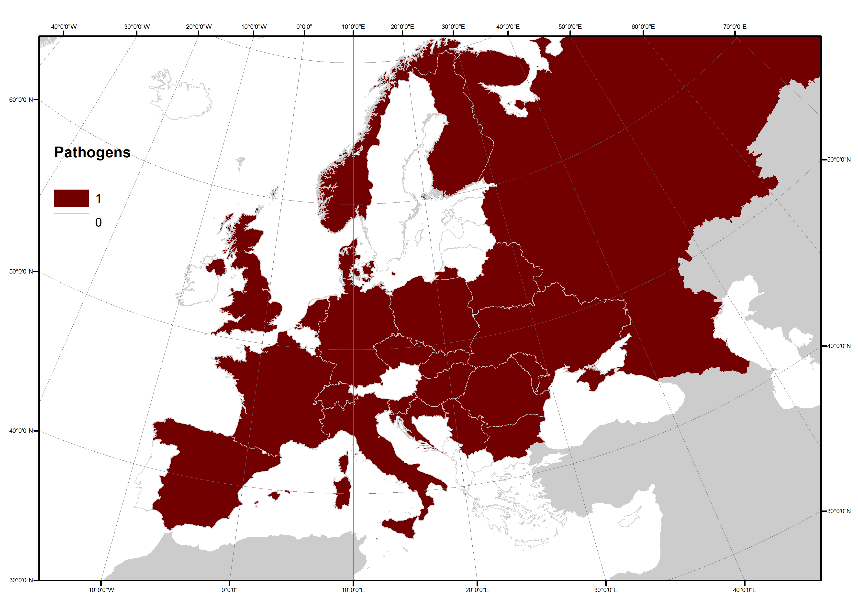


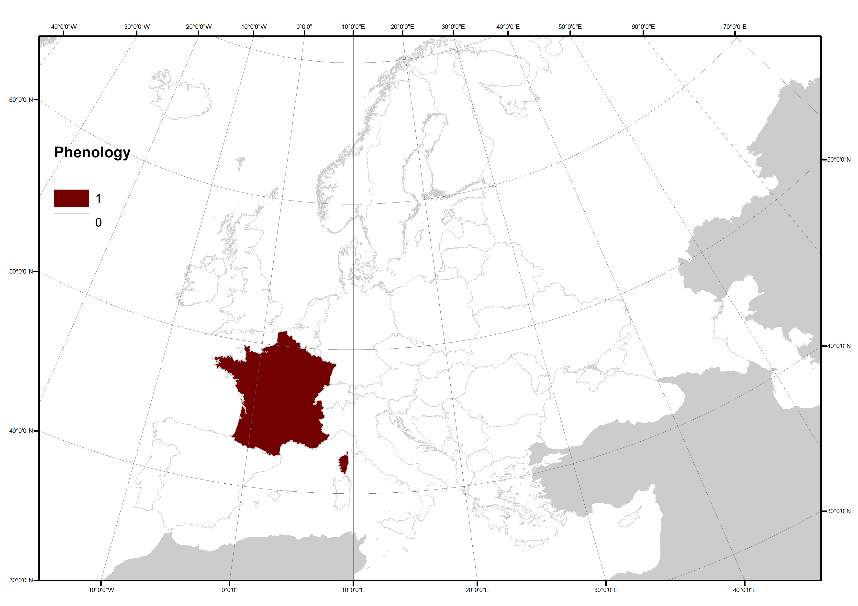

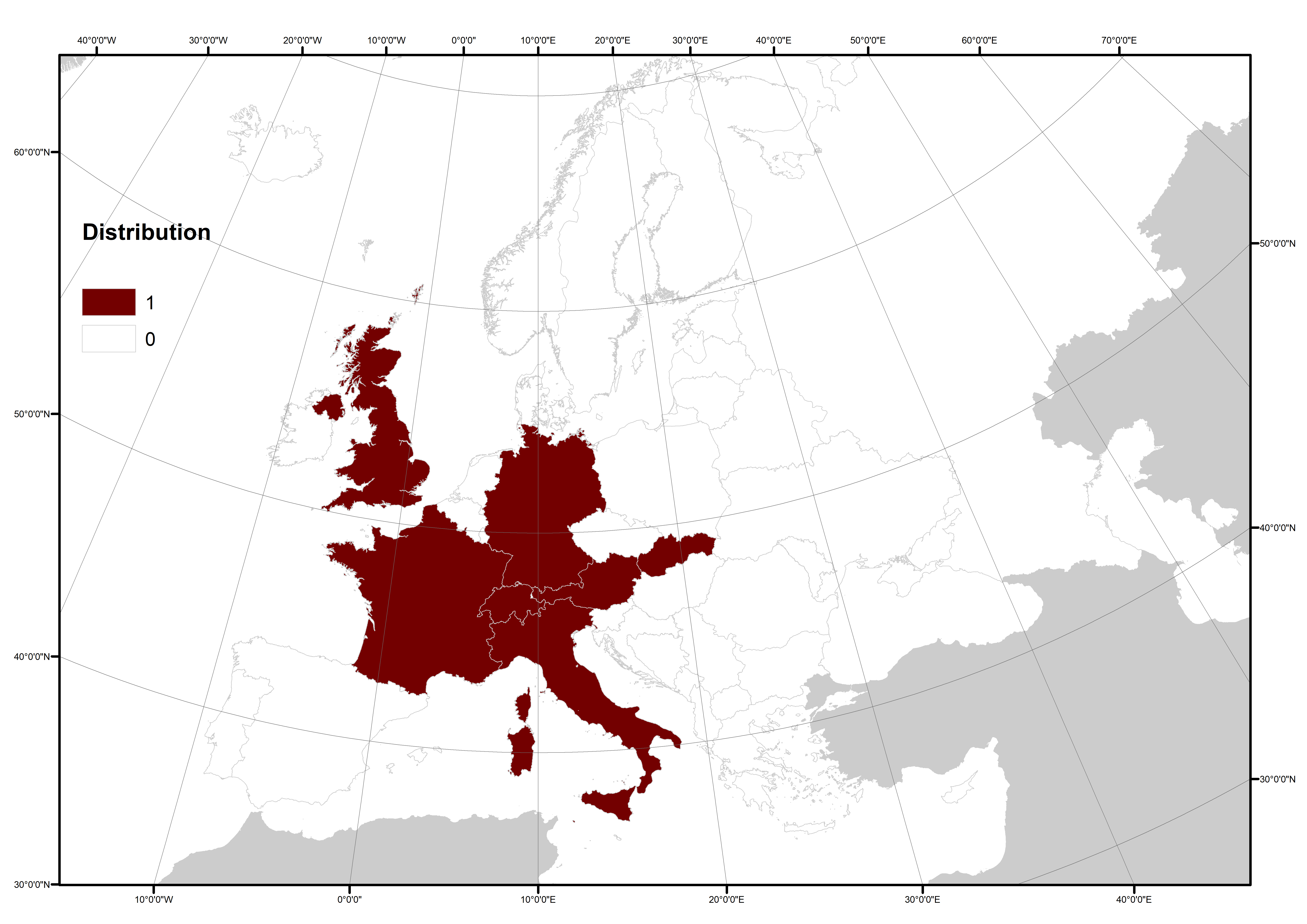


**Figure 1**. Geographic coverage of each trait category (genetic composition, physiology, morphology, acoustic signature, climatic associations, foraging habitat, roost type, diet, spatial behaviour, life history, pathogens, phenology, and distribution) provided at the country level. For sake of clarity and for highlighting gaps in geographic coverage, we did not consider in this map traits provided across a given species’ range or at regional level.

Supplementary Material 5. Individual-level morphological traits for France

Table available as excel file.
